# Neonatal Nutrition and Brain Structure at 7 Years in Children Born Very Preterm

**DOI:** 10.1001/jamanetworkopen.2024.56080

**Published:** 2025-01-24

**Authors:** Tanya Poppe, Anna C. Tottman, Greg D. Gamble, Yannan Jiang, Andrew E. Silva, Linda Nguyen, Jane E. Harding, Jane M. Alsweiler, Benjamin Thompson

**Affiliations:** 1 Department of Optometry and Vision Science, University of Auckland, Auckland, New Zealand; 2 Liggins Institute, University of Auckland, Auckland, New Zealand; 3 School of Optometry and Vision Science, University of Waterloo, Waterloo, Ontario, Canada; 4 Department of Pediatrics: Child and Youth Health, University of Auckland, Auckland, New Zealand; 5 Newborn Services, National Women’s Health, Auckland City Hospital, Auckland, New Zealand; 6 Centre for Eye and Vision Research, Hong Kong

## Abstract

**Importance:**

Neonatal protein intake following very preterm birth has long lasting effects on brain development. However, it is uncertain whether these effects are associated with improved or impaired brain maturation.

**Objective:**

To assess the association of neonatal protein intake following very preterm birth with brain structure at 7 years of age.

**Design, Setting, and Participants:**

This cohort study involved children born very preterm before or after a change in neonatal intensive care unit nutritional protocol that increased protein intake at the National Women’s Hospital in Auckland, New Zealand. The children completed magnetic resonance imaging (MRI) scanning at 7 years. There were 128 children who were initially eligible. MRI data were ineligible for analysis if excessive head motion or clinical brain abnormalities were present. Data were collected from July 2012 to January 2016, and data analysis took place from January 2017 to March 2024.

**Exposure:**

Neonatal intensive care unit nutritional protocol. Those who were born before the protocol change took place (July 2005 to December 2006) were in the old protocol group, while those who were born after the protocol change (January 2007 to October 2008) were in the new protocol group. Observers were blind to participant grouping.

**Main Outcomes and Measures:**

All actual enteral and parenteral intakes of protein, fat, energy, and breast milk for days 1 to 7 and days 1 to 14, and growth velocity to postnatal day 28 were calculated for each infant. Preplanned outcomes were group comparisons between regional brain volumes and diffusion parameters of major white matter tracts along with analyses with both groups combined exploring associations of nutrition with brain metrics.

**Results:**

Data from 99 children were analyzed, including 42 in the old protocol group (26 female [55%]; mean [SD] gestational age at birth, 27 [2] weeks) and 57 in the new protocol group (27 female [47%]; mean [SD] gestational age at birth, 26 [2] weeks). Protein intake differed between the groups at both 7 days (old protocol: mean [SD] intake, 17 [2] g/kg^−1^; new protocol: mean [SD] intake, 21 [2] g/kg^−1^) and 14 days after birth (old protocol: mean [SD] intake, 41 [6] g/kg^−1^; new protocol: mean [SD] intake, 45 [7] g/kg^−1^). The new protocol group had smaller brain volume as a percentage of intercranial volume than the old protocol group (mean [SD], 80% [4%] vs 86% [7%]) but absolute brain volumes were similar. The new protocol group had significantly thinner lateral occipital and lateral parietal cortices than the old protocol group. With both groups combined, those with greater protein, fat, energy, and breast milk intake had more mature diffusion tensor metrics (higher fractional anisotropy and less diffusion) across multiple tracts, although this finding did not reach statistical significance for every tract.

**Conclusions and Relevance:**

In this cohort of children born very preterm, children with greater neonatal protein intake had a more mature profile of brain metrics assessed with MRI at 7 years of age. These results contribute to the ongoing evaluation of optimal nutrition for infants born very preterm and suggest that the protein intake experienced by the new protocol group may promote brain maturation in a way that is still observable at 7 years of age.

## Key Points

**Question:**

Is protein intake following very preterm birth associated with brain volume and white matter microstructure at 7 years of age?

**Findings:**

In this cohort study of 99 seven-year-old children born very preterm (<30 weeks’ gestation or <1500 g birth weight), 57 children who received a higher protein nutrition protocol had significantly thinner lateral occipital and parietal cortices than 42 children who received standard nutrition. With both groups combined, those with higher protein intake had more mature diffusion tensor metrices (higher fractional anisotropy and less diffusion).

**Meaning:**

These findings suggest that protein intake following very preterm birth may influence brain maturation at 7 years of age.

## Teaser

This cohort study analyzes associations of nutrition intake with brain structure at 7 years of age among children born very preterm in New Zealand.

## Introduction

Preterm birth affects 10% of infants worldwide.^1^ Up to 80% of infants born at or before 32 weeks of gestation (very preterm) have a dysmaturation of brain development coupled with encephalopathy of prematurity.^2,3^ These conditions have been associated with neurobehavioral differences in childhood that persist into adolescence and adulthood.^4^ Most infants born very preterm require enteral and parenteral nutritional support over the first weeks after birth, but the ideal amount and composition is not yet known.

The association of neonatal nutrition with brain structure in infants born very preterm has most commonly been studied at term equivalent age (TEA). Increased energy and lipid intakes for very preterm infants in the first 2 weeks after birth to meet European Society of Paediatric Gastroenterology and Nutrition (ESPGHAN) guidelines (2011-2013)^5^ was associated with a lower incidence of brain dysmaturation and brain lesions at TEA.^6^ In the same cohort, protein, energy, lipid, and carbohydrate intake were all positively associated with cerebral volume at TEA.^7^ Lipid and energy intakes were also positively associated with basal ganglia and cerebellar volumes and with white mater tract fractional anisotropy (FA) at TEA.^7^ In a different cohort, higher amino acid (parenteral) and protein (enteral) intakes in infants with very low birth weight were associated with lower mean diffusivity (MD) in the superior longitudinal fasciculus at TEA.^8^ Variations in brain structure at TEA associated with neonatal nutrition are also associated with neurodevelopmental outcomes in early childhood^7^ or adolescence,^9^ suggesting that the impact of neonatal nutrition on brain development has lasting consequences.

Neonatal intensive care units (NICUs) frequently review and update their neonatal nutrition practices, particularly in line with ESPGHAN recommended nutrient intakes.^10,11^ In 2007, the NICU at National Women’s Hospital, Auckland, New Zealand, updated its neonatal nutrition guidelines with the intention of increasing early protein intake and decreasing fluid intake. This study involves a cohort of children born very preterm who experienced 1 of 2 nutrition protocols because of this change in nutrition practice. Neurodevelopmental and visual outcomes for this cohort have been reported previously.^12,13,14,15^ The number of infants below the 10th percentile for weight at their time of hospital discharge was reduced after the change.^12^ At 18 months’ corrected age, the change in nutrition was not associated with changes in Bayley III cognitive, motor, or language scores, but mean enteral protein intake in weeks 1 and 2 after birth were positively associated with cognitive and motor scores.^12^

There were no differences in body composition or neurodevelopmental outcomes between the 2 groups at 7 years of age. However, higher parenteral protein intake in postnatal week 1 was associated with an increased rate of cerebral palsy.^13^ Conversely, resting state magnetic resonance imaging (MRI) measurements indicated that greater protein intake in week 1 along with lower carbohydrate and fat intake were associated with stronger connectivity between thalamic networks and the default mode network.^14^ Connectivity strength between thalamic and default mode networks was, in turn, positively associated with scores on standardized tests of processing speed and visual processing. In addition, clinical and psychophysical measures of visual function indicated that higher protein intake was associated with better motion coherence thresholds, an index of dorsal cortical visual processing stream function, but a lower rate of positive binocular vision outcomes.^15^ Therefore, the composition of nutrition after very preterm birth appears to have a complex association with brain development assessed by MRI and neurocognitive outcomes at school age. To further explore these associations, we assessed whether changes in early neonatal nutrition in this cohort of very preterm infants were associated with brain tissue volumes and white matter microstructure at 7 years of age.

## Methods

### Participants

This cohort study was approved by the Northern B Health and Disability Ethics Committee and followed the Strengthening the Reporting of Observational Studies in Epidemiology (STROBE) reporting guideline. In January of 2007, an update to the neonatal intensive care unit nutrition protocol for very preterm infants at the National Women’s Hospital in Auckland, New Zealand led to increased protein, energy, fat, and mineral intakes and decreased total fluid intake.^12^ The study cohort consisted of children born from July 2005 to December 2006 before the change in protocol, who formed the old protocol group, and those born after the change (January 2007 to October 2008) who constituted the new protocol group. Children born within this time were eligible for this study at 7 years’ corrected age if they were born before 30 weeks’ gestational age or had a birth weight of less than 1500 g and were cared for in the level 3 NICU at National Women’s Hospital. Children were excluded if they were admitted to the NICU after 24 hours of age, had a major congenital malformation, ultrasonography evidence of a large parenchymal hemorrhagic infarction, were discharged before day 7, or died before 7 years of age. All actual enteral and parenteral intakes of protein, fat, energy, and breast milk for days 1 to 7 and days 1 to 14 and growth velocity to postnatal day 28 were calculated for each infant.^13^ Parents or caregivers provided informed, written consent for their children to participate, and children provided informed verbal assent for each assessment. Maternal ethnicity was self-reported and followed the New Zealand Standard Classification, as required by the ethics committee. Ethnicity categories included African, Asian, European, Māori, and Pacific Islander.

### MRI Data Acquisition

MRI data collection took place from July 2012 to January 2016. MRI scans were acquired using a Siemens MAGNETOM Skyra 3T using a 32-channel head coil at the Centre for Advanced MRI, University of Auckland, New Zealand. The maximum total scan time, including set up, was 1 hour and 15 minutes. Head movement was minimized by headphones and foam cushions within the head coil. Earplugs (3M Nitro; 32 dB attenuation) were used to minimize scan noise and children watched a movie of their choice during the scan. The T1 (longitudinal relaxation time) images were acquired using a magnetization-prepared rapid gradient echo pulse sequence with a repetition time (TR) of 2000 milliseconds, an echo time (TE) of 3510 milliseconds, an inversion time (TI) of 1010 milliseconds, 0.85 mm^3^ isometric voxels, field of view (FOV) of 210 mm, and anterior-to-posterior phase encoding, sagittally acquired. T2 (transverse relaxation time) images were acquired using a sampling perfection with application optimized contrast sequence with a TR of 3200 milliseconds, TE of 327 milliseconds, 0.9 mm^3^ isometric voxels, FOV of 210 mm, and anterior-to-posterior phase encoding, sagittally acquired. Diffusion weighted imaging volumes were acquired using a pediatric sequence that used 26 noncontiguous directions and 1 b0 image. B-values were pseudorandomized between 5 to 1200. The sequence had a TR of 10800 milliseconds, TE of 97 milliseconds, and 2 mm^3^ isotropic voxels with a FOV of 224 mm. Susceptibility weighted images, fluid attenuated inversion recovery, a functional MRI visual task, and resting state functional MRI images were also part of the protocol but are not reported here.

### Statistical Analysis

The classification of tissue types, structures, and cortical labeling was performed using the recon-all pipeline of FreeSurfer 6.0 image analysis suite (Laboratories for Computational Neuroimaging ).^16^ Data were inspected and manually corrected if necessary and resubmitted to the recon-all pipeline. Diffusion data were preprocessed in the Functional, Structural, and Diffusion MRI Software Library version 6.0.1 (FMRIB Analysis Group) before automatic reconstruction of 10 (2 bilateral and 8 unilateral) major white matter tracts using global probabilistic tractography with the anatomical priors of the tracts constrained by underlying anatomy tool in FreeSurfer 6.0.^17^ Cortical parcellations were combined to limit the number of comparisons.^18^ Statistical analysis was based on a written statistical analysis plan agreed upon by the steering group before data were unmasked. Data were analyzed using general linear models in the R-based software JASP version 0.18.3 (University of Amsterdam), with planned adjustment for sex, birth weight *z* score, gestation at birth (complete weeks), socioeconomic status (New Zealand Deprivation Index [NZDep]^19^), Clinical Risk Index for Babies, version 2 (CRIB II) score, and multiplicity if any of these baseline variables differed by more than 10% between the old protocol and new protocol groups. Comparisons were also adjusted for brain volume except for comparisons of brain volume, total intracranial volume, and percentage of intracranial volume that is brain volume. Inspection of histograms of the residuals and homoscedasticity and QQ plots were performed. Statistical analyses included between-group comparisons and testing for associations of brain structure with nutrition variables using combined data from both groups. Overall, there was no evidence for important violations of the assumptions underlying the use of linear models. Both uncorrected and multiple-comparisons corrected (false discovery rate) *P* values are presented. A *P* value less than .05 was considered statistically significant. Data analysis occurred from January 2017 to March 2024.

## Results

Of 128 children assessed at 7 years of age, 12 declined MRI, 16 of 115 MRI scans were excluded because of excess head motion, and 1 was excluded for dilated ventricles. In total, datasets from 99 children were included in this analysis, with 42 children in the old protocol group (26 female [55%]; mean [SD] gestational age at birth, 27 [2] weeks) and 57 children in the new protocol group (27 female [47%]; mean [SD] gestational age at birth, 26 [2] weeks) (Figure).

**Figure.**
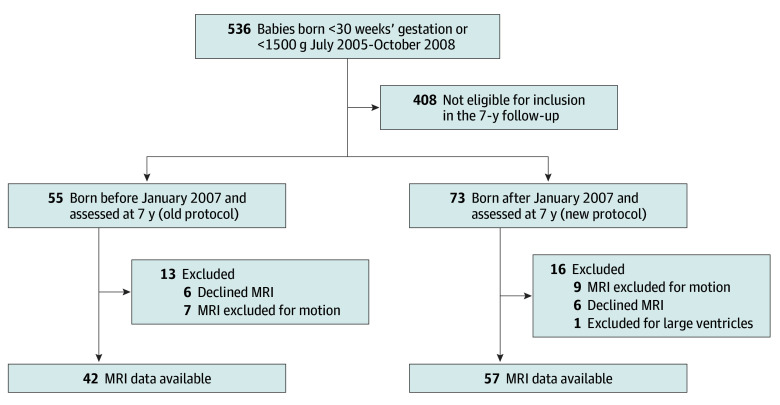
Participant Flow Diagram MRI indicates magnetic resonance imaging.

The children in the old protocol and new protocol groups who completed MRI measurements had similar maternal and neonatal characteristics (Table 1). However, sex, birth weight *z* score, and NZDep scores differed by more than 10% between groups and were adjusted for in general linear models. Protein intake differed, with children in the new protocol group receiving more protein than the old protocol group from days 1 to 7 (mean [SD] intake, 21 [2] g/kg^−1^ vs 17 [2] g/kg^−1^) and 1 to 14 (mean [SD] protein intake, 45 [7] g/kg^−1^ vs 41 [6] g/kg^−1^) but there were no other significant differences in neonatal nutrient intakes between groups (Table 1). At 7 years of age, there were no anthropometric differences between the old protocol and new protocol groups (Table 2).

**Table 1.** Maternal and Neonatal Characteristics and Neonatal Nutrition of Children Who Had Magnetic Resonance Imaging Data

Characteristic	Participants, No. (%) (N = 99)		*P* value^a^
	Old nutritional protocol (n = 42)	New nutritional protocol (n = 57)	
Maternal characteristics			
Maternal diabetes	3 (7)	2 (4)	.41
Maternal ethnicity			
African	1 (2)	0	.67
Asian	4 (10)	7 (12)	
European	23 (55)	25 (44)	
Māori	11(26)	14 (25)	
Pacific Islander	3 (7)	11 (19)	
Antenatal steroids	39 (93)	49 (86)	.28
Multiple pregnancy	9 (21)	17 (30)	.34
Neonatal characteristics			
Gestational age at birth, mean (SD), wk	27 (2)	26 (2)	.11
Born at <30 wk	38 (90)	56 (98)	.16
Birth weight, mean (SD), g	933 (251)	913 (192)	.65
Birth weight <1500 g	41 (98)	57 (100)	.42
Gestational age <30 wk and birth weight <1500 g	37 (88)	56 (98)	.08
Birth weight, mean (SD), *z* score	−0.1 (0.8)	0.1 (0.9)	.14
Sex			
Male	19 (45)	30 (53)	.47
Female	26 (55)	27 (47)	
CRIB II score, mean (SD)	10 (3)	10 (2)	.43
Intraventricular hemorrhage			
None	37 (88)	45 (79)	.23
Grade I	2 (5)	4 (7)	
Grade II	3 (7)	4 (7)	
Grade III	0	1 (2)	
Grade IV	0	3 (5)	
Periventricular leukomalacia	1 (2)	1 (2)	.83
Necrotizing enterocolitis	1 (2)	2 (4)	.75
Early infection	0	1 (2)	.68
Late infection	7 (17)	11 (19)	.72
Major neonatal surgery	1 (2)	7 (12)	.07
Hypoglycemia	12 (29)	15 (26)	.80
Birth length, mean (SD), cm	35 (3)	35 (3)	.93
Birth length mean (SD), *z* score	0 (1)	0 (1)	.43
Head circumference at birth, mean (SD), cm	25 (2)	24 (2)	.17
Head circumference at birth, mean (SD) *z* score	0 (1)	0 (1)	.38
Head circumference at 36 wk, mean (SD), cm	31 (1)	31 (1)	.77
Head circumference at 36 wk, mean (SD) *z* score	−1 (1)	−1 (1)	.87
Growth velocity at 28 d , mean (SD) g/kg/d^−1^	11 (4)	11 (4)	.72
Bronchopulmonary dysplasia	10 (24)	20 (35)	.23
Postmenstrual age at discharge, mean (SD), wk	39 (2)	40 (3)	.06
Neonatal nutrition characteristics			
Total protein, mean (SD), g/kg^−1^			
Days 1-7	17 (2)	21 (2)	<.001
Days 1-14	41 (6)	45 (7)	.004
Total fat, mean (SD), g/kg^−1^			
Days 1-7	25 (6)	25 (5)	.84
Days 1-14	70 (12)	68 (15)	.42
Total energy, mean (SD), kcal/kg^−1^			
Days 1-7	596 (76)	571 (65)	.08
Days 1-14	1509 (203)	1430 (256)	.10
Total breast milk, mean (SD), mL/kg^−1^			
Days 1-7	260 (190)	212 (140)	.15
Days 1-14	1132 (518)	993 (487)	.18

Abbreviation: CRIB II, Clinical Risk Index for Babies, version 2.

^a^ Group comparisons were made using independent samples *t* tests for continuous measures and Fischer test of proportions for categorical measures.

**Table 2.** Anthropometry and Neurodevelopmental Outcomes at 7 Years of Children Who Had Magnetic Resonance Imaging Data

Outcome	Participants, No. (%) (N = 99)		*P* value
	Old nutritional protocol (n = 42)	New nutritional protocol (n = 57)	
Corrected age at assessment, mean (SD), y	7 (0)	7 (0)	NA
NZDep score , mean (SD)	5 (3)	6 (3)	.07
Weight, mean (SD), kg	24 (6)	26 (8)	.18
Weight, mean (SD), *z* score	0 (1)	0 (1)	.12
Height, mean (SD), cm	123 (6)	125 (6)	.16
Height, mean (SD), *z* score	0 (1)	0 (1)	.13
Head circumference, mean (SD), cm	52 (2)	52 (2)	.58
Head circumference , mean (SD), *z* score	−1 (1)	−1 (2)	.48
No neurodevelopmental impairment	25 (60)	36 (63)	.69
Neurodevelopmental impairment^a^	17 (40)	21 (37)	.71
WISC FSIQ score <85	16 (38)	13 (23)	.10
MABC-2 total score ≤5th percentile	8 (19)	15 (26)	.40
Cerebral palsy	0	6 (11)	.09
Blind	0	0	NA
Deaf	1 (2)	1 (2)	0.83

Abbreviations: NA, not applicable; NZDep, New Zealand Socioeconomic Deprivation Index; MABC-2, Movement Assessment Battery for Children version 2; WISC FSIQ, Wechsler Intelligence Scale for Children full scale intelligence quotient.

^a^ Neurodevelopmental impairment was defined as WISC FSIQ less than 85, MABC-2 total score in the fifth percentile or lower, cerebral palsy, blindness, or deafness (requiring aids).

Children in the new protocol group had a smaller brain volume as a percentage of intracranial volume than children in the old protocol group (mean [SD], 80% [4%] vs 86% [7%]), but there was no difference in total brain volume between groups (Table 3). The lateral-occipital and parietal cortices were bilaterally thinner in the new protocol group, although the left lateral parietal difference did not survive multiple comparisons correction (Table 4). There were no consistent patterns of differences between groups in white matter diffusivity metrics (eTable 1 in Supplement 1), although the new protocol group exhibited lower mean and radial diffusivities in bilateral cingulum angular bundles and the left uncinate fasciculus as well as lower axial diffusivity in the right superior longitudinal fasciculus and forceps minor compared with the old protocol group.

**Table 3.** Global and Regional Brain Volumes at 7 Years of Age

Measurement	Volume, mean, (SD) cm^3^		Mean difference (95% CI)^a^		*P* value	Corrected *P* value (FDR)
	Old protocol	New protocol	Unadjusted	Adjusted		
Total intracranial volume^b^	1438.70 (162.85)	1518.33 (140.83)	79.63 (18.88 to 140.39)	65.39 (10.51 to 120.28)	.02	.21
Brain volume, % of intracranial volume^b^	85.78 (5.74)	79.94 (3.90)	−5.84 (−7.78 to −3.91)	−5.75 (−7.77 to −3.73)	<.001	<.001
Brain volume^b^	1236.90 (127.71)	1213.37 (120.30)	−23.53 (−73.37 to 26.31)	−34.46 (−79.73 to 10.82)	.13	.79
Brain tissue volume	1218.65 (126.86)	1191.56 (118.60)	−27.09 (−76.39 to 22.21)	−2.3 (−11.70 to 7.10)	.63	.79
Cerebrospinal fluid	0.86 (0.29)	0.84 (0.30)	−0.02 (−0.14 to 0.10)	0.00 (-0.12 to 0.13)	.96	.96
Ventricles	18.12 (13.69)	21.57 (26.54)	3.44 (−5.45 to 12.34)	2.16 (−7.03 to 11.36)	.64	.79
Total grey matter	797.94 (81.64)	775.17 (77.60)	−22.78 (−54.8 to 9.24)	−6.00 (−16.28 to 4.29)	.25	.79
Cortical grey matter	625.99 (68.68)	606.6 (67.57)	−19.39 (−46.85 to 8.07)	−5.16 (−15.09 to 4.77)	.30	.79
Left hemisphere cortical grey matter	313.42 (34.25)	303.58 (33.38)	−9.85 (−23.47 to 3.78)	−2.70 (−7.81 to 2.42)	.30	.79
Right hemisphere cortical grey matter	312.57 (34.60)	303.02 (34.53)	−9.55 (−23.49 to 4.40)	-2.46 (-7.61 to 2.69)	.34	.79
Subcortical grey matter	55.44 (4.64)	55.24 (4.55)	−0.20 (−2.05 to 1.65)	0.46 (−0.75 to 1.68)	.45	.79
Nucleus accumbens	1.06 (0.17)	1.07 (0.17)	0.01 (−0.06 to 0.07)	0.01 (−0.05 to 0.07)	.76	.81
Amygdala	3.26 (0.32)	3.14 (0.38)	−0.12 (−0.26 to 0.02)	-0.07 (-0.18 to 0.05)	.25	.79
Caudate nucleus	7.22 (1.12)	7.37 (0.99)	0.15 (−0.27 to 0.58)	0.31 (−0.03 to 0.66)	.08	.61
Hippocampus	7.86 (0.70)	7.64 (0.81)	−0.21 (−0.52 to 0.10)	−0.15 (−0.42 to 0.12)	.28	.79
Pallidum	3.57 (0.41)	3.76 (0.46)	0.19 (0.01 to 0.37)	0.21 (0.05 to 0.37)	.01	.17
Putamen	9.87 (1.05)	9.96 (1.26)	0.09 (−0.38 to 0.57)	0.09 (-0.36 to 0.53)	.70	.81
Thalamus	13.95 (1.48)	13.80 (1.12)	−0.15 (−0.67 to 0.37)	0.09 (-0.26 to 0.44)	.61	.79
Ventral diencephalon	7.32 (0.67)	7.12 (0.67)	−0.19 (−0.46 to 0.08)	−0.09 (−0.31 to 0.13)	.42	.79
Cerebral white matter	395.14 (49.95)	390.69 (45.11)	−4.45 (−23.51 to 14.61)	3.25 (−5.14 to 11.65)	.44	.79
Left hemisphere cerebral white matter	198.42 (25.23)	196.30 (22.39)	−2.12 (−11.66 to 7.42)	1.57 (−2.69 to 5.83)	.47	.79
Right hemisphere cerebral white matter	196.72 (24.81)	194.39 (23.05)	−2.33 (−11.94 to 7.28)	1.68 (−2.68 to 6.04)	.45	.79
Corpus callosum	3.33 (0.61)	3.24 (0.65)	−0.09 (−0.35 to 0.16)	−0.06 (−0.31 to 0.18)	.60	.79
Anterior corpus callosum	0.85 (0.19)	0.86 (0.25)	0.01 (−0.09 to 0.10)	0.03 (−0.06 to 0.12)	.51	.79
Central corpus callosum	0.60 (0.16)	0.56 (0.16)	−0.04 (−0.11 to 0.02)	−0.04 (−0.11 to 0.03)	.25	.79
Midanterior corpus callosum	0.63 (0.20)	0.58 (0.16)	−0.04 (−0.11 to 0.03)	-0.04 (-0.10 to 0.03)	.29	.79
Midposterior corpus callosum	0.48 (0.11)	0.49 (0.12)	0.01 (−0.04 to 0.05)	0.00 (-0.04 to 0.05)	.84	.87
Posterior corpus callosum	0.77 (0.17)	0.75 (0.22)	−0.02 (−0.10 to 0.06)	-0.02 (−0.10 to 0.06)	.58	.79
Brainstem	16.80 (1.89)	16.73 (1.74)	-0.07 (-0.80 to 0.66)	0.11 (-0.52 to 0.74)	.74	.81
Cerebellar cortex	113.92 (13.91)	111.24 (14.88)	−2.67 (−8.52 to 3.17)	−0.83 (−5.82 to 4.16)	.74	.81
Cerebellar white matter	25.30 (3.58)	25.33 (4.86)	0.04 (−1.72 to 1.80)	0.41 (−1.29 to 2.11)	.63	.79
Choroid plexus	0.65 (0.30)	0.70 (0.34)	0.05 (−0.08 to 0.18)	0.04 (−0.09 to 0.18)	.52	.79

Abbreviation: FDR, false discovery rate.

^a^ Each mean difference was adjusted for sex, birth weight *z* score, New Zealand Socioeconomic Deprivation Index, and brain volume.

^b^ Measures not adjusted for brain volume. Brain tissue volume is volume excluding ventricles.

**Table 4.** Regional Cortical Thickness at 7 Years of Age

Measurement	Cortical thickness, mean (SD), mm		Mean difference (95% CI)^a^		*P* value	Corrected *P* value (FDR)
	Old protocol	New protocol	Unadjusted	Adjusted		
Left hemisphere	2.99 (0.15)	2.91 (0.17)	−0.08 (−0.15 to −0.02)	−0.06 (−0.12 to 0.01)	.09	.28
Right hemisphere	3.00 (0.15)	2.92 (0.16)	−0.08 (−0.14 to −0.01)	−0.06 (−0.12 to 0.01)	.08	.28
Cingulate						
Left	9.25 (0.65)	9.10 (0.67)	−0.15 (−0.42 to 0.12)	−0.10 (−0.36 to 0.17)	.47	.59
Right	9.17 (0.66)	9.03 (0.67)	−0.14 (−0.41 to 0.13)	−0.14 (−0.42 to 0.13)	.30	.50
Inferior-frontal						
Left	6.19 (0.39)	6.12 (0.39)	−0.07 (−0.23 to 0.09)	−0.03 (−0.19 to 0.13)	.73	.79
Right	6.27 (0.44)	6.08 (0.37)	−0.20 (−0.36 to −0.04)	−0.15 (−0.31 to 0.02)	.08	.28
Midfrontal						
Left	9.73 (0.64)	9.50 (0.62)	−0.22 (−0.48 to 0.03)	−0.17 (−0.42 to 0.09)	.19	.43
Right	9.63 (0.70)	9.42 (0.71)	−0.21 (−0.49 to 0.08)	−0.13 (−0.41 to 0.16)	.39	.57
Orbital-frontal						
Left	9.60 (0.71)	9.71 (0.62)	0.12 (−0.15 to 0.38)	0.17 (−0.10 to 0.44)	.22	.43
Right	9.73 (0.72)	9.66 (0.68)	−0.08 (−0.36 to 0.21)	−0.01 (−0.30 to 0.29)	.97	.97
Superior-frontal						
Left	3.46 (0.19)	3.41 (0.22)	−0.05 (−0.13 to 0.04)	−0.02 (−0.10 to 0.07)	.67	.77
Right	3.40 (0.19)	3.35 (0.22)	−0.05 (−0.13 to 0.03)	−0.03 (−0.11 to 0.06)	.48	.59
Precentral gyrus						
Left	2.97 (0.22)	2.89 (0.28)	−0.08 (−0.19 to 0.02)	−0.05 (−0.15 to 0.05)	.35	.53
Right	2.93 (0.29)	2.87 (0.25)	−0.06 (−0.17 to 0.05)	−0.04 (−0.15 to 0.07)	.46	.59
Postcentral gyrus						
Left	2.62 (0.25)	2.53 (0.20)	−0.09 (−0.18 to 0.00)	−0.06 (−0.15 to 0.03)	.21	.43
Right	2.62 (0.23)	2.50 (0.21)	−0.12 (−0.21 to −0.03)	−0.09 (−0.18 to 0.00)	.05	.28
Insula						
Left	3.49 (0.19)	3.40 (0.20)	−0.10 (−0.17 to −0.02)	−0.07 (−0.15 to 0.01)	.08	.28
Right	3.50 (0.22)	3.43 (0.20)	−0.07 (−0.16 to 0.01)	−0.06 (−0.14 to 0.03)	.19	.43
Isthmus						
Left	2.87 (0.27)	2.80 (0.23)	−0.08 (−0.17 to 0.02)	−0.06 (−0.16 to 0.04)	.23	.43
Right	2.78 (0.24)	2.80 (0.29)	0.02 (−0.09 to 0.13)	0.06 (−0.04 to 0.17)	.24	.43
Lateral-occipital						
Left	2.54 (0.21)	2.35 (0.19)	−0.19 (−0.27 to −0.11)	−0.16 (−0.24 to −0.09)	<.001	<.001
Right	2.66 (0.17)	2.48 (0.19)	−0.19 (−0.26 to −0.11)	−0.17 (−0.24 to −0.10)	<.001	<.001
Medial-occipital						
Left	4.73 (0.35)	4.61 (0.45)	−0.12 (−0.29 to 0.05)	−0.07 (−0.24 to 0.105)	.45	.59
Right	4.65 (0.39)	4.65 (0.39)	0.00 (−0.16 to 0.16)	0.05 (−0.12 to 0.21)	.58	.68
Lateral-parietal						
Left	5.75 (0.36)	5.48 (0.41)	−0.27 (−0.43 to −0.11)	−0.21 (−0.36 to −0.06)	.01	.07
Right	5.75 (0.39)	5.45 (0.41)	−0.30 (−0.46 to −0.14)	−0.25 (−0.41 to −0.09)	<.001	.03
Medial-parietal						
Left	8.59 (0.56)	8.37 (0.58)	−0.23 (−0.45 to 0.01)	−0.12 (−0.35 to 0.108)	.30	.50
Right	8.43 (0.46)	8.34 (0.48)	−0.09 (−0.28 to 0.10)	−0.03 (−0.22 to 0.16)	.76	.80
Supramarginal						
Left	2.99 (0.21)	2.85 (0.25)	−0.14 (−0.23 to −0.047)	−0.10 (−0.19 to −0.01)	.03	.20
Right	3.01 (0.22)	2.89 (0.25)	−0.12 (−0.21 to −0.02)	−0.091 (−0.186 to 0.003)	.06	.28
Inferior-temporal						
Left	6.37 (0.37)	6.24 (0.32)	−0.13 (−0.27 to 0.01)	−0.09 (−0.23 to 0.05)	.23	.43
Right	6.36 (0.36)	6.32 (0.34)	−0.04 (−0.18 to 0.10)	0.00 (−0.14 to 0.14)	.97	.97
Lateral-temporal						
Left	6.02 (0.44)	5.78 (0.41)	−0.24 (−0.42 to −0.07)	−0.17 (−0.34 to 0.01)	.06	.28
Right	6.23 (0.38)	6.03 (0.41)	−0.21 (−0.37 to −0.05)	−0.13 (−0.28 to 0.02)	.10	.29
Medial-temporal						
Left	10.56 (1.02)	10.65 (0.73)	0.09 (−0.26 to 0.437)	0.177 (−0.187 to 0.542)	.34	.53
Right	10.62 (1.02)	10.77 (0.70)	0.15 (−0.20 to 0.49)	0.27 (−0.07 to 0.61)	.12	.33
Superior-temporal						
Left	6.27 (0.51)	6.17 (0.42)	−0.09 (−0.28 to 0.09)	−0.03 (−0.21 to 0.15)	.73	.79
Right	6.39 (0.47)	6.27 (0.46)	−0.12 (−0.30 to 0.07)	−0.08 (−0.26 to 0.11)	.42	.59

Abbreviation: FDR, false discovery rate.

^a^ Each regression was adjusted for sex, birth weight *z* score, New Zealand Socioeconomic Deprivation Index, and brain volume.

Preplanned general linear models exploring the associations of protein, fat, energy, and breast milk intakes with measures of brain structure did not reveal many statistically significant associations, but 3 general findings emerged (eTables 2-4 in Supplement 1). First, when data from both groups were combined, those with less protein intake had smaller brain volume relative to intracranial volume. Conversely, those with greater intakes of fat, energy, and breast milk all had greater brain volume relative to intracranial volume at days 1 to 7 (eTable 1 in Supplement 1). Second, those with less protein intake for days 1 to 7 had smaller cortical thickness in almost every brain area measured, whereas those with greater fat, energy, and breast milk intakes had greater cortical thickness in most brain areas measured (eTable 3 in Supplement 1). Third, those with greater intakes of protein, fat, energy, and breast milk in days 1 to 7 and days 1 to 14 had more mature white matter microstructure in almost every tract studied (higher FA and lower MD, axial diffusivity, and radial diffusivity) (eTable 4 in Supplement 1).

## Discussion

In this cohort study, we explored the association of early nutrition following preterm birth with later brain development by collecting structural MRI data from a cohort of 7-year-old children born very preterm and compared the new protocol group, who had a higher protein intake during the first 14 days after birth due to a change in nutrition practices within the NICU, with the old protocol group. Intakes of fat, energy, and breast milk were similar between the 2 groups. MRI revealed that the old protocol group had a lower percentage of intracranial volume that was brain volume (greater extracerebral space) and thinner cortex in the lateral occipital and parietal regions than the old protocol group. When data from both groups were combined, those with greater early neonatal protein intake had greater extracerebral space combined with a thinner cortex, and those with greater intakes of protein, fat, energy, and breast milk had more mature white matter microstructure metrics, but these findings were not statistically significant. These results are consistent with our previous studies of this cohort, which indicated enhanced cortico-thalamic connectivity^14^ and dorsal stream function at age 7 years.^15^ However, these apparently positive effects of increased protein were accompanied by an increased risk for cerebral palsy^13^ and reduced binocular vision.^15^ In addition, a recent randomized trial of additional parenteral amino acid supplementation in in the first 5 days after birth in infants with extremely low birth weight found that the higher intake group was more likely to have moderate or severe disability at 2 years of age.^35^

The greater extracerebral space that we observed in the new protocol group is a well-documented preterm phenotype^20^ associated with younger gestational age at birth.^21,22^ However, we did not observe a difference in brain volume between the new protocol and old protocol groups, suggesting that the difference in extracerebral space was due to greater intracranial volume in the new protocol group. In other words, the groups had similar size brains, but the new protocol group had a larger space inside the cranium. The mechanism through which increased protein may influence intracranial volume is unclear. However, neonatal nutrition has previously been associated with intracranial volume variability, without an associated increase in brain volume, in seven-year-old children born very preterm.^23^ In particular, more breastfeeding was associated with greater intracranial volume but not with brain volume.^23^ Interestingly, our analysis of both groups combined suggested the opposite, whereby those with a greater intake of breast milk had more of the intracranial space being occupied by brain.

Cortical thinning is a normal maturational process, beginning from ages 5 to 8 years depending on the brain area.^24^ Preterm birth is associated with changes in cortical thinning, possibly indicating delayed or abnormal brain maturation. For example, preterm infants have a thicker cortex at TEA than full term–born controls^25^ and cortical thinning occurs later in children born preterm than in those born at full term.^26^ However, adolescents born before 28 weeks’ gestation had thinner cortices in the middle temporal and posterior parietal cortices than full term–born controls.^27^ These patterns are challenging to interpret because cross-sectional group differences in cortical thickness could represent restricted cortical growth (ie, a thinner cortex to start with),^28^ delayed maturation,^26^ or a combination of both effects. We propose that the thinner lateral occipital and parietal cortex observed in the new protocol group compared with the old protocol group is indicative of a more advanced pattern of cortical maturation because resting state MRI data from a subset of the same cohort revealed that increased neonatal protein intake was positively associated with the strength of functional connectivity between the thalamus and the default mode network.^14^ A positive association of protein intake with cortical maturation is consistent with these functional connectivity results.

We observed that infants with less protein intake had smaller cortical thickness throughout the brain for the cohort overall, and a pronounced between-group difference for the lateral occipital cortex, with the new protocol group having significantly thinner cortex in this region. The lateral occipital cortex may be particularly sensitive to the effects of preterm birth. Children born very preterm had greater lateral occipital cortical gray matter volume^18^ and cortical thickness^29^ at 7 years of age than term controls, possibly indicating delayed maturation. However, individuals born with a very low birth weight and term controls who had reached adolescence did not differ in lateral-occipital thickness, suggesting that these differences may decrease as the brain matures.^30^

The lateral occipital cortex includes several areas specialized for visual processing that project to the parietal and temporal lobes to form the dorsal and ventral cortical processing streams.^31^ We have previously reported lower (better) motion coherence thresholds in the new protocol group than the old protocol group at 7 years of age.^15^ Motion coherence is a psychophysical index of processing within the dorsal cortical stream^32,33^ which projects from the primary visual cortex via motion sensitive visual area middle temporal to the parietal lobe.^31^ In agreement with these behavioral findings, the lateral parietal area was also significantly thinner in the new protocol group compared with the old protocol group. This pattern of results further supports the possibility that thinner cortex is associated with more advanced brain maturation in this cohort.

Children born preterm have smaller deep gray matter volumes than their full term–born counterparts at 7 years of age^34^ and enriched early neonatal nutrition is associated with greater deep gray matter volumes at TEA.^7^ At 7 years of age, the new protocol group had larger pallidi than the old protocol group, but this finding was not significant after false discovery rate correction. Those with greater early protein intake had greater thalamic volume and those with greater early fat, energy, and breast milk intake had larger nucleus accumbens and putamen volumes. Our results suggest that early neonatal nutrition may impact the development of critical deep brain structures that are evident at midchildhood.

There were no statistically significant group differences in white matter tract characteristics that were consistent across all 4 diffusion tensor imaging metrics, although there were higher FA and lower diffusivities in major white matter tracts in the new protocol group compared with the old protocol group. In addition, when both groups were combined, those with greater intakes of protein, fat, energy, and breast milk had more mature white matter tract metrics (increased FA and reduced MD, axial diffusivity, and radial diffusivity) in almost every tract studied. Together, these results highlight the importance of neonatal nutrition for white matter tract maturation, even at age 7 years.

### Strengths and Limitations

Strengths of this study include a relatively large cohort of children born very preterm with detailed, individually calculated information about early nutritional intakes and long-term follow-up to 7 years of age. MRI data included both structural and diffusion tensor imaging measures and data quality was high with only 16 of 115 scans excluded because of excessive head movement. Study limitations include the fact that not all eligible children participated in the MRI component of the study and that children were not randomized to the 2 different nutrition groups. Therefore, it is unclear whether other differences in standard of care may have contributed to the study results.

## Conclusions

In this cohort study of infants born very preterm, structural and diffusion tensor imaging measures indicated that children born very preterm who had increased protein intake had enhanced brain maturation at 7 years. Perinatal protein intake can have a long-term impact on brain development in children born very preterm, but whether the impact is positive or negative may depend on the domain of development (eg, sensory vs motor) and the absolute amount and nature of the protein.

## Notes

Supplement 1. **eTable 1.** Diffusion Tensor Imaging Parameters of Major White Matter Tracts at Seven Years of Age of Children in the LowPro and HiPro Groups **eTable 2.** Associations Between the Intake of Protein, Fat, Total Energy and Breastmilk and Brain Volumes for the Whole Cohort **eTable 3.** Associations Between the Intake of Protein, Fat, Total Energy and Breastmilk and Cortical Thickness for the Whole Cohort **eTable 4.** Associations Between the Intake of Protein, Fat, Total Energy and Breastmilk and Diffusion Tensor Imaging Metrics of White Matter Microstructure for the Whole Cohort

Supplement 2.
Nonauthor Collaborators

Supplement 3.
Data Sharing Statement
