# Supplementary material for: Neonatal Nutrition and Brain Structure at 7 Years in Children Born Very Preterm
Source: JAMA Netw Open. 2025 Jan 24;8(1):e2456080. doi: 10.1001/jamanetworkopen.2024.56080 (PMC11762234; doi:10.1001/jamanetworkopen.2024.56080)
Supplement: Supplement 1. — eTable 1. Diffusion Tensor Imaging Parameters of Major White Matter Tracts at Seven Years of Age of Children in the LowPro and HiPro Groups eTable 2. Associations Between the Intake of Protein, Fat, Total Energy and Breastmilk and Brain Volumes for the Whole Cohort eTable 3. Associations Between the Intake of Protein, Fat, Total Energy and Breastmilk and Cortical Thickness for the Whole Cohort eTable 4. Associations Between the Intake of Protein, Fat, Total Energy and Breastmilk and Diffusion Tensor Imaging Metrics of White Matter Microstructure for the Whole Cohort [file jamanetwopen-e2456080-s001.pdf]

Supplemental Online Content

Poppe T, Tottman A, Gamble GD. Neonatal nutrition and brain structure at 7 years in children born very perterm. *JAMA Netw. Open*. 2025;8(1):e2456080. doi:10.1001/jamanetworkopen.2024.56080

eTable 1. Diffusion Tensor Imaging Parameters of Major White Matter Tracts at Seven Years of Age of Children in the LowPro and HiPro Groups

eTable 2. Associations Between the Intake of Protein, Fat, Total Energy and Breastmilk and Brain Volumes for the Whole Cohort

eTable 3. Associations Between the Intake of Protein, Fat, Total Energy and Breastmilk and Cortical Thickness for the Whole Cohort

eTable 4. Associations Between the Intake of Protein, Fat, Total Energy and Breastmilk and Diffusion Tensor Imaging Metrics of White Matter Microstructure for the Whole Cohort

This supplemental material has been provided by the authors to give readers additional information about their work.

© 2025 Poppe T et al. *JAMA Network Open*.

eTable 1. Diffusion tensor imaging parameters of major white matter tracts at seven years of age of children in the LowPro and HiPro groups.

Fractional anistropy		OldPro Mean (SD)	NewPro Mean (SD)	Unadjusted Mean Difference (95% CI)	Adjusted Mean Difference (95% CI)	*p*	Corrected *p* (FDR)
Forceps major		0.44 (0.06)	0.46 (0.07)	0.021 (-0.012, 0.054)	0.021 (-0.013, 0.055)	0.22	0.57
Forceps minor		0.44 (0.05)	0.43 (0.05)	-0.012 (-0.036, 0.011)	-0.017 (-0.041, 0.008)	0.18	0.57
Anterior thalamic radiation	left	0.43 (0.03)	0.43 (0.03)	-0.002 (-0.015, 0.012)	-0.001 (-0.014, 0.013)	0.90	0.90
	right	0.43 (0.04)	0.43 (0.02)	0.002 (-0.012, 0.017)	0.002 (-0.012, 0.017)	0.75	0.88
Cingulum angular bundle	left	0.30 (0.04)	0.32 (0.05)	0.015 (-0.006, 0.037)	0.015 (-0.007, 0.037)	0.17	0.57
	right	0.29 (0.04)	0.30 (0.04)	0.007 (-0.014, 0.028)	0.010 (-0.012, 0.031)	0.38	0.76
Cingulate gyrus	left	0.50 (0.06)	0.49 (0.05)	-0.016 (-0.041, 0.010)	-0.015 (-0.041, 0.012)	0.27	0.61
	right	0.46 (0.05)	0.45 (0.05)	-0.005 (-0.028, 0.018)	-0.003 (-0.027, 0.021)	0.81	0.88
Corticospinal tract	left	0.55 (0.04)	0.55 (0.03)	0.004 (-0.012, 0.019)	0.002 (-0.012, 0.017)	0.74	0.88
	right	0.53 (0.05)	0.53 (0.05)	-0.003 (-0.025, 0.019)	-0.004 (-0.027, 0.019)	0.72	0.88
Inferior longitudinal fasciculus	left	0.46 (0.04)	0.46 (0.05)	-0.004 (-0.024, 0.017)	-0.004 (-0.025, 0.018)	0.73	0.88
	right	0.46 (0.05)	0.46 (0.04)	-0.006 (-0.027, 0.015)	-0.004 (-0.025, 0.018)	0.74	0.88
Superior longitudinal fasciculus parietal	left	0.43 (0.04)	0.42 (0.04)	-0.015 (-0.033, 0.003)	-0.014 (-0.032, 0.004)	0.13	0.57
	right	0.42 (0.04)	0.42 (0.03)	-0.003 (-0.019, 0.012)	-0.002 (-0.017, 0.014)	0.83	0.88
Superior longitudinal fasciculus temporal	left	0.46 (0.04)	0.44 (0.03)	-0.015 (-0.030, 0.000)	-0.013 (-0.028, 0.002)	0.08	0.57
	right	0.45 (0.03)	0.44 (0.03)	-0.010 (-0.024, 0.005)	-0.009 (-0.024, 0.005)	0.21	0.57
Uncinate fasciculus	left	0.37 (0.03)	0.38 (0.03)	0.009 (-0.007, 0.025)	0.011 (-0.004, 0.026)	0.16	0.57
	right	0.38 (0.04)	0.38 (0.04)	0.003 (-0.014, 0.020)	0.005 (-0.013, 0.022)	0.60	0.88
				Unadjusted	Adjusted Mean		

© 2025 Poppe T et al. *JAMA Network Open*.

Mean diffusivity x10^{-4}mm^{2}s^{-1}		OldPro Mean (SD)	NewPro Mean (SD)	Mean Difference (95% CI)	Difference (95% CI)	*p*	Corrected *p* (FDR)
Forceps major		8.91 (0.49)	8.86 (0.46)	-0.047 (-0.292, 0.198)	-0.048 (-0.290, 0.195)	0.70	0.78
Forceps minor		8.49 (0.37)	8.38 (0.35)	-0.116 (-0.295, 0.064)	-0.074 (-0.257, 0.109)	0.42	0.59
Anterior thalamic radiation	left	8.01 (0.43)	7.87 (0.27)	-0.147 (-0.314, 0.020)	-0.142 (-0.311, 0.026)	0.10	0.29
	right	8.02 (0.36)	7.85 (0.25)	-0.174 (-0.318, -0.031)	-0.168 (-0.311, -0.026)	0.02	0.13
Cingulum angular bundle	left	9.67 (0.69)	9.31 (0.44)	-0.364 (-0.630, -0.098)	-0.339 (-0.612, -0.066)	0.02	0.13
	right	9.56 (0.61)	9.23 (0.50)	-0.325 (-0.583, -0.068)	-0.320 (-0.586, -0.055)	0.02	0.13
Cingulate gyrus	left	8.13 (0.58)	8.06 (0.32)	-0.074 (-0.290, 0.141)	-0.089 (-0.313, 0.134)	0.43	0.59
	right	8.09 (0.56)	8.02 (0.37)	-0.073 (-0.291, 0.145)	-0.096 (-0.312, 0.120)	0.38	0.59
Corticospinal tract	left	7.93 (0.53)	7.77 (0.31)	-0.158 (-0.358, 0.042)	-0.144 (-0.347, 0.059)	0.16	0.36
	right	8.06 (0.54)	7.92 (0.39)	-0.136 (-0.355, 0.082)	-0.129 (-0.356, 0.099)	0.26	0.53
Inferior longitudinal fasciculus	left	8.78 (0.55)	8.72 (0.34)	-0.058 (-0.270, 0.153)	-0.052 (-0.272, 0.167)	0.64	0.76
	right	8.79 (0.47)	8.66 (0.34)	-0.135 (-0.325, 0.055)	-0.152 (-0.342, 0.038)	0.11	0.29
Superior longitudinal fasciculus parietal	left	7.92 (0.67)	7.91 (0.35)	-0.009 (-0.255, 0.236)	-0.007 (-0.262, 0.248)	0.96	0.96
	right	7.92 (0.46)	7.89 (0.31)	-0.028 (-0.210, 0.154)	-0.050 (-0.235, 0.135)	0.59	0.76
Superior longitudinal fasciculus temporal	left	8.11 (0.43)	8.11 (0.32)	0.005 (-0.172, 0.182)	-0.013 (-0.195, 0.169)	0.89	0.94
	right	8.01 (0.45)	7.95 (0.35)	-0.061 (-0.248, 0.125)	-0.089 (-0.277, 0.099)	0.35	0.59
Uncinate fasciculus	left	8.63 (0.33)	8.46 (0.30)	-0.168 (-0.315, -0.021)	-0.157 (-0.307, -0.008)	0.04	0.18
	right	8.66 (0.30)	8.51 (0.33)	-0.145 (-0.292, 0.002)	-0.141 (-0.287, 0.004)	0.06	0.20

Axial diffusivity x10^{-4}mm^{2}s^{-1}		OldPro Mean (SD)	NewPro Mean (SD)	Unadjusted Mean Difference (95% CI)	Adjusted Mean Difference (95% CI)	*p*	Corrected *p* (FDR)
Forceps major		13.70 (1.02)	13.88 (0.82)	0.186 (-0.280, 0.652)	0.180 (-0.284, 0.643)	0.44	0.47

© 2025 Poppe T et al. *JAMA Network Open*.

Forceps minor		13.02 (0.67)	12.66 (0.61)	-0.354 (-0.672, -0.035)	-0.356 (-0.684, -0.029)	0.03	0.20
Anterior thalamic radiation	left	12.04 (0.61)	11.81 (0.38)	-0.237 (-0.471, -0.003)	-0.217 (-0.460, 0.025)	0.08	0.20
	right	12.06 (0.57)	11.82 (0.39)	-0.237 (-0.461, -0.014)	-0.229 (-0.462, 0.004)	0.05	0.20
Cingulum angular bundle	left	12.86 (1.06)	12.57 (0.69)	-0.293 (-0.706, 0.119)	-0.258 (-0.685, 0.170)	0.23	0.35
	right	12.61 (0.82)	12.26 (0.85)	-0.345 (-0.737, 0.046)	-0.316 (-0.719, 0.087)	0.12	0.25
Cingulate gyrus	left	13.21 (0.94)	12.89 (0.60)	-0.321 (-0.685, 0.042)	-0.335 (-0.714, 0.044)	0.08	0.20
	right	12.52 (0.87)	12.36 (0.72)	-0.164 (-0.535, 0.207)	-0.173 (-0.558, 0.211)	0.37	0.47
Corticospinal tract	left	13.39 (0.76)	13.17 (0.46)	-0.213 (-0.502, 0.075)	-0.206 (-0.507, 0.095)	0.18	0.29
	right	13.36 (0.82)	13.09 (0.44)	-0.273 (-0.574, 0.029)	-0.272 (-0.587, 0.043)	0.09	0.20
Inferior longitudinal fasciculus	left	13.62 (0.71)	13.51 (0.60)	-0.105 (-0.413, 0.204)	-0.104 (-0.423, 0.215)	0.52	0.52
	right	13.62 (0.58)	13.35 (0.64)	-0.272 (-0.561, 0.018)	-0.271 (-0.566, 0.023)	0.07	0.20
Superior longitudinal fasciculus parietal	left	11.86 (0.81)	11.66 (0.41)	-0.196 (-0.491, 0.098)	-0.176 (-0.484, 0.132)	0.26	0.36
	right	11.77 (0.53)	11.69 (0.42)	-0.077 (-0.299, 0.145)	-0.092 (-0.323, 0.138)	0.43	0.47
Superior longitudinal fasciculus temporal	left	12.46 (0.56)	12.26 (0.39)	-0.201 (-0.424, 0.022)	-0.201 (-0.433, 0.031)	0.09	0.20
	right	12.24 (0.52)	12.02 (0.43)	-0.216 (-0.436, 0.004)	-0.255 (-0.481, -0.029)	0.03	0.20
Uncinate fasciculus	left	12.21 (0.46)	12.08 (0.47)	-0.129 (-0.347, 0.089)	-0.089 (-0.303, 0.124)	0.41	0.47
	right	12.37 (0.46)	12.19 (0.46)	-0.173 (-0.388, 0.041)	-0.150 (-0.365, 0.065)	0.17	0.29

Radial diffusivity x10^{-4}mm^{2}s^{-1}		OldPro Mean (SD)	NewPro Mean (SD)	Unadjusted Mean Difference (95% CI)	Adjusted Mean Difference (95% CI)	*p*	Corrected *p* (FDR)
Forceps major		6.52 (0.60)	6.35 (0.71)	-0.163 (-0.508, 0.181)	-0.161 (-0.513, 0.191)	0.36	0.81
Forceps minor		6.23 (0.50)	6.23 (0.47)	0.004 (-0.238, 0.245)	0.067 (-0.180, 0.314)	0.59	0.87
Anterior thalamic radiation	left	6.00 (0.44)	5.90 (0.31)	-0.103 (-0.277, 0.072)	-0.105 (-0.277, 0.067)	0.23	0.68
	right	6.00 (0.40)	5.86 (0.27)	-0.143 (-0.301, 0.015)	-0.138 (-0.292, 0.016)	0.08	0.35

© 2025 Poppe T et al. *JAMA Network Open*.

Cingulum angular bundle	left	8.08 (0.64)	7.68 (0.50)	-0.399 (-0.665, -0.133)	-0.379 (-0.652, -0.107)	0.01	0.13
	right	8.03 (0.64)	7.71 (0.49)	-0.315 (-0.580, -0.051)	-0.323 (-0.597, -0.048)	0.02	0.20
Cingulate gyrus	left	5.59 (0.67)	5.64 (0.46)	0.049 (-0.217, 0.316)	0.033 (-0.240, 0.307)	0.81	0.89
	right	5.87 (0.57)	5.84 (0.46)	-0.027 (-0.267, 0.213)	-0.058 (-0.295, 0.180)	0.63	0.87
Corticospinal tract	left	5.20 (0.53)	5.07 (0.36)	-0.130 (-0.340, 0.079)	-0.113 (-0.317, 0.091)	0.27	0.70
	right	5.41 (0.59)	5.34 (0.56)	-0.068 (-0.335, 0.199)	-0.057 (-0.332, 0.218)	0.68	0.88
Inferior longitudinal fasciculus	left	6.36 (0.61)	6.33 (0.47)	-0.035 (-0.289, 0.219)	-0.026 (-0.291, 0.239)	0.84	0.89
	right	6.38 (0.60)	6.31 (0.42)	-0.066 (-0.307, 0.174)	-0.093 (-0.336, 0.150)	0.45	0.81
Superior longitudinal fasciculus parietal	left	5.95 (0.66)	6.03 (0.43)	0.084 (-0.174, 0.342)	0.078 (-0.186, 0.341)	0.56	0.87
	right	5.99 (0.50)	5.99 (0.36)	-0.003 (-0.207, 0.201)	-0.029 (-0.232, 0.174)	0.78	0.89
Superior longitudinal fasciculus temporal	left	5.93 (0.48)	6.04 (0.36)	0.108 (-0.087, 0.303)	0.082 (-0.113, 0.277)	0.41	0.81
	right	5.90 (0.49)	5.91 (0.39)	0.016 (-0.189, 0.222)	-0.006 (-0.210, 0.199)	0.96	0.96
Uncinate fasciculus	left	6.84 (0.39)	6.65 (0.35)	-0.188 (-0.361, -0.014)	-0.191 (-0.366, -0.016)	0.03	0.20
	right	6.80 (0.39)	6.67 (0.39)	-0.131 (-0.314, 0.053)	-0.137 (-0.323, 0.049)	0.15	0.52

Each regression was adjusted for sex, birthweight z-score, and New Zealand socioeconomic deprivation index. Data are presented as group means. AD= axial diffusivity, RD= radial diffusivity, MD= mean diffusivity, FA= fractional anisotropy.

© 2025 Poppe T et al. *JAMA Network Open*.

eTable 2. Associations between the intake of protein, fat, total energy and breastmilk and brain volumes for the whole cohort.

Predicted Variable	Predictor: Protein intake (g.kg^{-1}.day^{-1})						Predictor: Fat intake (g.kg^{-1}.day^{-1})						Predictor: Energy intake (g.kg^{-1}.day^{-1})						Predictor: Breastmilk intake (ml.kg^{-1}day^{-1})					
	Days 1-7			Days 1-14			Days 1-7			Days 1-14			Days 1-7			Days 1-14			Days 1-7			Days 1-14		
	Beta	p	Corrected p (FDR)	Beta	p	Corrected p (FDR)	Beta	p	Corrected p (FDR)	Beta	p	Corrected p (FDR)	Beta	p	Corrected p (FDR)	Beta	p	Corrected p (FDR)	Beta	p	Corrected p (FDR)	Beta	p	Corrected p (FDR)
Total Intracranial Volume	4.293	0.33	0.75	1.717	0.40	0.81	-3.545	0.16	0.46	-1.002	0.31	0.63	-0.359	0.06	0.25	-0.056	0.34	0.71	-0.134	0.11	0.42	-0.038	0.17	0.46
Relative Brain Volume to Intracranial Volume	-0.484	0.01	0.44	-0.076	0.41	0.81	0.229	0.04	0.36	0.095	0.03	0.49	0.025	3E-3	0.10	0.006	0.03	0.47	0.011	3E-3	0.08	0.003	0.01	0.13
Brain Volume	-3.310	0.35	0.75	0.242	0.88	0.92	0.360	0.86	0.90	0.551	0.49	0.64	0.068	0.67	0.81	0.037	0.43	0.71	0.047	0.49	0.75	0.016	0.47	0.69
Brain Tissue Volume (excluding ventricles)	0.116	0.87	0.94	-0.191	0.57	0.81	0.751	0.07	0.36	0.222	0.17	0.63	0.062	0.05	0.23	0.009	0.36	0.71	0.025	0.07	0.42	0.008	0.06	0.35
Cerebrospinal fluid	-0.006	0.57	0.90	0.002	0.64	0.81	-0.005	0.35	0.51	-0.002	0.43	0.63	-4.1E-4	0.34	0.57	-4.1E-5	0.75	0.86	-2.8E-4	0.13	0.42	-1.1E-4	0.07	0.35
Ventricles	-0.141	0.84	0.94	0.179	0.59	0.81	-0.745	0.07	0.36	-0.220	0.16	0.63	-0.062	0.05	0.23	-0.009	0.35	0.71	-0.024	0.07	0.42	-0.008	0.06	0.35
Total Grey Matter	-0.518	0.52	0.87	-0.271	0.46	0.81	0.499	0.28	0.49	0.174	0.33	0.63	0.048	0.17	0.37	0.007	0.49	0.74	0.020	0.19	0.49	0.004	0.45	0.69
Cortical Grey Matter	-0.571	0.46	0.87	-0.207	0.56	0.81	0.535	0.23	0.46	0.163	0.34	0.63	0.049	0.15	0.35	0.008	0.44	0.71	0.018	0.24	0.50	0.002	0.67	0.77
Left Hemisphere Cortical Grey Matter	-0.191	0.63	0.92	-0.093	0.61	0.81	0.260	0.25	0.48	0.095	0.28	0.63	0.028	0.11	0.28	0.005	0.37	0.71	0.009	0.23	0.50	0.001	0.60	0.77
Right Hemisphere Cortical Grey Matter	-0.380	0.34	0.75	-0.114	0.54	0.81	0.276	0.23	0.46	0.068	0.44	0.63	0.021	0.24	0.44	0.003	0.55	0.77	0.008	0.27	0.51	0.001	0.76	0.81
Subcortical Grey Matter	-0.035	0.71	0.93	0.036	0.41	0.81	0.065	0.23	0.46	0.022	0.30	0.63	-0.001	0.88	0.91	0.001	0.43	0.71	0.002	0.27	0.51	0.001	0.09	0.36
Nucleus Accumbens	0.004	0.48	0.87	0.003	0.15	0.81	0.007	0.02	0.34	0.003	0.01	0.30	4.5E-4	0.04	0.23	1.4E-4	0.03	0.47	1.5E-4	0.11	0.42	5.0E-5	0.11	0.40
Amygdala	-0.013	0.14	0.75	-0.004	0.30	0.81	-0.005	0.33	0.51	-0.002	0.22	0.63	-4.4E-4	0.25	0.44	-1.2E-4	0.31	0.71	-6.5E-5	0.70	0.86	-6.2E-5	0.26	0.46
Caudate Nucleus	0.013	0.62	0.92	0.016	0.20	0.81	0.002	0.88	0.90	0.004	0.52	0.64	-2.7E-4	0.82	0.90	2.8E-4	0.44	0.71	-1.0E-4	0.85	0.96	8.0E-5	0.64	0.77
Hippocampus	-0.033	0.12	0.75	-0.017	0.08	0.81	-0.010	0.40	0.56	-0.004	0.37	0.63	-0.002	0.10	0.28	-3.6E-4	0.19	0.71	-1.8E-4	0.65	0.83	-2.4E-5	0.86	0.88
Pallidum	0.014	0.27	0.75	0.007	0.24	0.81	0.011	0.13	0.45	0.003	0.36	0.63	8.1E-5	0.89	0.91	9.0E-5	0.60	0.80	3.8E-4	0.12	0.42	1.5E-4	0.06	0.35
Putamen	-0.002	0.96	0.96	-0.002	0.92	0.92	0.045	0.02	0.34	0.011	0.16	0.63	0.002	0.15	0.35	4.4E-4	0.33	0.71	0.002	0.01	0.17	0.001	1E-3	0.05
Thalamus	-0.002	0.95	0.96	0.029	0.02	0.60	0.024	0.12	0.45	0.008	0.20	0.63	0.001	0.57	0.76	0.001	0.10	0.61	0.001	0.20	0.49	2.0E-4	0.25	0.46
Ventral Diencephalon	-0.018	0.28	0.75	-0.002	0.83	0.92	0.004	0.67	0.79	0.002	0.55	0.64	-0.001	0.42	0.60	1.8E-5	0.94	0.97	5.2E-5	0.87	0.96	6.7E-5	0.53	0.74
Cerebral White Matter	0.660	0.31	0.75	0.133	0.66	0.81	0.366	0.33	0.51	0.079	0.59	0.64	0.025	0.38	0.58	0.004	0.66	0.82	0.007	0.59	0.79	0.005	0.25	0.46
Left Hemisphere Cerebral White Matter	0.434	0.19	0.75	0.090	0.55	0.81	0.232	0.22	0.46	0.055	0.45	0.63	0.019	0.19	0.38	0.003	0.53	0.77	0.005	0.48	0.75	0.003	0.20	0.46
Right Hemisphere Cerebral White Matter	0.226	0.50	0.87	0.043	0.79	0.92	0.134	0.49	0.63	0.024	0.75	0.77	0.006	0.68	0.81	0.001	0.82	0.91	0.002	0.74	0.88	0.002	0.34	0.54
Corpus Callosum	0.030	0.11	0.75	0.013	0.13	0.81	0.008	0.47	0.63	0.005	0.22	0.63	0.001	0.08	0.27	3.8E-4	0.13	0.68	2.8E-4	0.44	0.74	1.6E-4	0.18	0.46
Anterior Corpus Callosum	0.008	0.27	0.75	0.006	0.07	0.81	0.001	0.82	0.90	0.002	0.15	0.63	2.2E-4	0.47	0.66	1.5E-4	0.09	0.61	-9.6E-6	0.94	0.96	5.4E-5	0.22	0.46
Central Corpus Callosum	0.007	0.19	0.75	4.6E-4	0.85	0.92	0.005	0.08	0.36	0.001	0.30	0.63	0.001	0.01	0.10	6.3E-5	0.37	0.71	1.6E-4	0.12	0.42	3.6E-5	0.28	0.47
Mid-anterior Corpus Callosum	0.008	0.11	0.75	0.003	0.21	0.81	0.004	0.16	0.46	0.002	0.10	0.63	0.001	0.02	0.19	1.2E-4	0.09	0.61	1.4E-4	0.15	0.45	5.6E-5	0.09	0.36
Mid-posterior Corpus Callosum	0.005	0.18	0.75	0.002	0.24	0.81	0.001	0.77	0.89	0.001	0.50	0.64	1.4E-4	0.37	0.58	4.4E-5	0.37	0.71	6.2E-5	0.38	0.68	2.9E-5	0.21	0.46
Posterior Corpus Callosum	0.002	0.76	0.94	0.002	0.55	0.81	-0.003	0.35	0.51	-0.001	0.56	0.64	-8.6E-5	0.75	0.86	8.5E-7	0.99	0.99	-7.6E-5	0.52	0.75	-1.7E-5	0.66	0.77
Brainstem	-0.007	0.89	0.94	0.003	0.91	0.92	0.003	0.91	0.91	0.006	0.60	0.64	-0.001	0.62	0.79	1.0E-4	0.87	0.93	-1.1E-4	0.91	0.96	1.8E-4	0.56	0.75
Cerebellar Cortex	0.136	0.72	0.93	-0.101	0.57	0.81	-0.111	0.62	0.76	-0.010	0.91	0.91	-0.001	0.96	0.96	-0.002	0.73	0.86	-3.9E-4	0.96	0.96	0.001	0.74	0.81
Cerebellar White Matter	-0.046	0.73	0.93	-0.047	0.44	0.81	-0.094	0.21	0.46	-0.024	0.41	0.63	-0.009	0.11	0.28	-0.002	0.27	0.71	-0.001	0.58	0.79	8.0E-6	0.99	0.99
Choroid Plexus	-0.002	0.85	0.94	0.004	0.37	0.81	-0.011	0.06	0.36	-0.002	0.41	0.63	-0.001	0.03	0.23	-6.0E-5	0.67	0.82	-4.5E-4	0.02	0.26	-8.8E-5	0.18	0.46

Linear regression models were adjusted for sex, birthweight z-score, New Zealand socioeconomic deprivation index. All analyses except for those involving metrics of Total Intracranial Volume, Relative Brain Volume to Intracranial Volume and Brain Volume were corrected for brain volume.

© 2025 Poppe T et al. *JAMA Network Open*.

eTable 3. Associations between the intake of protein, fat, total energy and breastmilk and cortical thickness for the whole cohort.

Predicted Variable		Predictor: Protein intake (g.kg^{-1}.day^{-1})						Predictor: Fat intake (g.kg^{-1}.day^{-1})						Predictor: Energy intake (g.kg^{-1}.day^{-1})						Predictor: Breastmilk intake (ml.kg^{-1}day^{-1})					
		Days 1-7			Days 1-14			Days 1-7			Days 1-14			Days 1-7			Days 1-14			Days 1-7			Days 1-14		
		Beta	p	Corrected p (FDR)	Beta	p	Corrected p (FDR)	Beta	p	Corrected p (FDR)	Beta	p	Corrected p (FDR)	Beta	p	Corrected p (FDR)	Beta	p	Corrected p (FDR)	Beta	p	Corrected p (FDR)	Beta	p	Corrected p (FDR)
Hemisphere Cortical Thickness	left	-0.005	0.29	0.76	1.1E-4	0.96	0.96	0.003	0.24	0.48	0.002	0.06	0.17	2.7E-4	0.21	0.56	1.1E-4	0.09	0.26	1.8E-4	0.05	0.19	5.1E-5	0.10	0.41
	right	-0.006	0.25	0.76	0.001	0.81	0.95	0.004	0.15	0.48	0.002	0.06	0.17	2.9E-4	0.18	0.56	1.1E-4	0.08	0.24	2.0E-4	0.03	0.19	4.8E-5	0.12	0.41
Pre-Central Gyrus	left	-0.002	0.78	0.88	0.004	0.24	0.85	0.006	0.16	0.48	0.004	0.04	0.17	0.001	0.12	0.45	2.3E-4	0.02	0.14	3.1E-4	0.04	0.19	7.9E-5	0.11	0.41
	right	-0.005	0.55	0.81	0.005	0.24	0.85	0.004	0.45	0.56	0.004	0.05	0.17	3.0E-4	0.41	0.71	2.3E-4	0.03	0.14	1.0E-4	0.53	0.59	3.7E-5	0.49	0.56
Post-Central Gyrus	left	-0.009	0.20	0.76	-0.007	0.02	0.30	4.5E-4	0.91	0.91	-0.001	0.58	0.71	2.4E-5	0.94	0.95	-8.0E-5	0.38	0.63	1.6E-4	0.24	0.32	4.3E-5	0.33	0.45
	right	-0.010	0.16	0.76	-0.004	0.17	0.85	0.005	0.21	0.48	0.001	0.54	0.68	3.6E-4	0.24	0.56	3.1E-5	0.74	0.84	2.8E-4	0.04	0.19	6.1E-5	0.17	0.41
Superiorfrontal	left	-0.006	0.38	0.81	0.003	0.32	0.85	0.005	0.16	0.48	0.003	0.02	0.11	1.8E-4	0.53	0.72	1.8E-4	0.04	0.14	1.9E-4	0.13	0.21	5.5E-5	0.18	0.41
	right	-0.006	0.34	0.76	0.003	0.25	0.85	0.005	0.22	0.48	0.003	0.02	0.11	2.0E-4	0.49	0.72	1.9E-4	0.02	0.14	1.8E-4	0.14	0.21	6.9E-5	0.10	0.41
Midfrontal	left	-0.033	0.09	0.71	-0.008	0.39	0.85	0.006	0.62	0.71	0.002	0.65	0.75	2.8E-4	0.75	0.84	1.2E-4	0.66	0.84	0.001	0.15	0.21	6.9E-5	0.58	0.63
	right	-0.006	0.77	0.88	0.010	0.31	0.85	0.015	0.24	0.48	0.007	0.14	0.29	0.001	0.27	0.57	4.7E-4	0.11	0.26	0.001	0.12	0.21	1.2E-4	0.38	0.48
Inferiorfrontal	left	-0.003	0.78	0.88	-0.001	0.87	0.95	0.003	0.72	0.78	0.003	0.31	0.49	3.2E-4	0.55	0.72	1.6E-4	0.34	0.58	2.5E-4	0.29	0.35	1.1E-4	0.15	0.41
	right	-0.018	0.17	0.76	-0.001	0.91	0.95	0.010	0.19	0.48	0.004	0.15	0.29	0.001	0.25	0.56	2.4E-4	0.16	0.32	0.001	0.03	0.19	1.2E-4	0.15	0.41
Orbitalfrontal	left	-1.3E-4	1.00	1.00	-0.002	0.83	0.95	0.005	0.67	0.75	-0.001	0.90	0.93	-3.9E-4	0.67	0.83	-9.9E-5	0.72	0.84	3.6E-4	0.37	0.44	2.9E-5	0.83	0.83
	right	-0.022	0.33	0.76	-0.001	0.91	0.95	0.011	0.38	0.55	0.002	0.66	0.75	1.1E-4	0.92	0.95	1.1E-4	0.71	0.84	0.001	0.18	0.24	8.9E-5	0.54	0.60
Superiortemporal	left	0.005	0.71	0.88	0.001	0.87	0.95	0.007	0.41	0.55	0.004	0.25	0.42	0.001	0.30	0.59	1.6E-4	0.40	0.63	4.4E-4	0.10	0.21	1.5E-4	0.10	0.41
	right	0.004	0.80	0.88	0.006	0.37	0.85	0.013	0.13	0.48	0.005	0.12	0.26	0.001	0.07	0.45	3.1E-4	0.11	0.26	0.001	0.04	0.19	1.3E-4	0.16	0.41
Lateraltemporal	left	-0.006	0.63	0.88	0.007	0.28	0.85	0.008	0.29	0.48	0.007	0.01	0.11	0.001	0.10	0.45	4.4E-4	0.01	0.11	3.8E-4	0.13	0.21	1.4E-4	0.11	0.41
	right	-0.007	0.54	0.81	0.014	0.01	0.30	0.010	0.12	0.48	0.007	4E-3	0.11	0.001	0.13	0.45	0.001	9E-4	0.03	3.7E-4	0.10	0.21	8.4E-5	0.26	0.41
Inferiortemporal	left	-0.010	0.34	0.76	-0.004	0.39	0.85	0.003	0.59	0.70	0.002	0.49	0.66	3.3E-4	0.49	0.72	6.2E-5	0.67	0.84	3.3E-4	0.12	0.21	5.8E-5	0.41	0.50
	right	-0.007	0.51	0.81	-0.003	0.61	0.95	0.011	0.08	0.48	0.002	0.34	0.52	4.2E-4	0.38	0.69	6.0E-5	0.68	0.84	4.8E-4	0.02	0.19	8.2E-5	0.23	0.41
Medialtemporal	left	-0.009	0.75	0.88	0.006	0.66	0.95	0.017	0.28	0.48	0.006	0.37	0.52	-4.7E-4	0.71	0.83	1.6E-4	0.66	0.84	0.001	0.11	0.21	2.4E-4	0.19	0.41
	right	0.010	0.72	0.88	0.003	0.81	0.95	0.020	0.19	0.48	0.002	0.72	0.76	7.1E-5	0.95	0.95	-4.7E-6	0.99	0.99	0.001	0.09	0.21	1.8E-4	0.29	0.41
Lateralparietal	left	-0.014	0.24	0.76	0.005	0.37	0.85	0.008	0.26	0.48	0.007	0.01	0.11	0.001	0.07	0.45	4.5E-4	4E-3	0.07	3.8E-4	0.09	0.21	1.4E-4	0.06	0.41
	right	-0.015	0.23	0.76	0.001	0.93	0.95	0.010	0.19	0.48	0.006	0.04	0.17	0.001	0.04	0.45	3.7E-4	0.03	0.14	4.5E-4	0.07	0.19	1.4E-4	0.08	0.41
Supramarginal	left	-0.002	0.81	0.88	-0.001	0.81	0.95	0.001	0.78	0.83	0.002	0.20	0.38	0.001	0.09	0.45	1.3E-4	0.17	0.33	9.0E-5	0.51	0.58	5.3E-5	0.24	0.41
	right	-0.007	0.33	0.76	0.005	0.18	0.85	0.004	0.32	0.49	0.004	0.02	0.11	5.0E-4	0.13	0.45	2.7E-4	0.01	0.07	1.6E-4	0.26	0.33	6.0E-5	0.20	0.41
Medialparietal	left	0.001	0.96	0.99	0.006	0.48	0.87	0.012	0.22	0.48	0.006	0.11	0.26	0.001	0.12	0.45	3.8E-4	0.11	0.26	0.001	0.09	0.21	1.2E-4	0.28	0.41
	right	-0.002	0.87	0.92	0.006	0.38	0.85	0.009	0.31	0.49	0.005	0.10	0.26	4.0E-4	0.53	0.72	2.8E-4	0.14	0.30	4.6E-4	0.10	0.21	1.0E-4	0.28	0.41
Lateraloccipital	left	-0.012	0.06	0.62	-0.001	0.66	0.95	0.006	0.12	0.48	0.003	0.04	0.17	0.001	0.06	0.45	1.7E-4	0.04	0.15	2.6E-4	0.03	0.19	6.3E-5	0.13	0.41
	right	-0.019	2E-3	0.08	-0.005	0.09	0.83	0.004	0.26	0.48	0.001	0.37	0.52	2.8E-4	0.32	0.60	6.0E-5	0.47	0.72	2.3E-4	0.06	0.19	4.3E-5	0.28	0.41
Medialoccipital	left	-0.010	0.46	0.81	0.004	0.51	0.88	0.011	0.13	0.48	0.006	0.05	0.17	3.6E-4	0.54	0.72	2.6E-4	0.13	0.29	0.001	0.04	0.19	1.0E-4	0.23	0.41
	right	-0.009	0.48	0.81	-0.002	0.72	0.95	0.006	0.42	0.55	0.002	0.51	0.66	-3.1E-4	0.58	0.73	5.6E-6	0.97	0.99	4.7E-4	0.05	0.19	1.0E-4	0.20	0.41
Cingulate	left	-0.013	0.51	0.81	-0.004	0.64	0.95	0.014	0.23	0.48	0.005	0.24	0.42	0.001	0.23	0.56	2.7E-4	0.32	0.58	0.001	0.17	0.24	2.3E-4	0.07	0.41
	right	-0.022	0.29	0.76	-0.004	0.71	0.95	-0.003	0.83	0.85	1.4E-4	0.98	0.98	-3.4E-4	0.72	0.83	6.5E-6	0.98	0.99	-6.5E-5	0.87	0.90	-2.9E-5	0.83	0.83
Isthmus	left	-0.015	0.05	0.62	-0.003	0.40	0.85	-0.003	0.42	0.55	0.001	0.71	0.76	-0.001	0.10	0.45	6.2E-6	0.95	0.99	6.2E-6	0.97	0.97	3.7E-5	0.44	0.52
	right	0.006	0.49	0.81	0.009	0.02	0.30	0.005	0.28	0.48	0.004	0.04	0.17	2.4E-4	0.51	0.72	2.4E-4	0.03	0.14	3.0E-4	0.06	0.19	9.1E-5	0.08	0.41
Insula	left	-0.011	0.06	0.62	-0.002	0.47	0.87	-0.005	0.16	0.48	-0.002	0.24	0.42	-3.5E-4	0.20	0.56	-5.3E-5	0.52	0.76	-2.0E-5	0.87	0.90	-3.6E-5	0.35	0.46
	right	-0.005	0.42	0.81	-0.002	0.43	0.86	-0.002	0.52	0.64	-0.001	0.67	0.75	-6.4E-5	0.83	0.90	-2.8E-5	0.75	0.84	3.0E-5	0.82	0.89	9.6E-6	0.82	0.83

Linear regression models were adjusted for sex, birthweight z-score, New Zealand socioeconomic deprivation index. All analyses except for those involving metrics of Total Intracranial Volume, Relative Brain Volume to Intracranial Volume and Brain Volume were corrected for brain volume.

© 2025 Poppe T et al. *JAMA Network Open*.

eTable 4. Associations between the intake of protein, fat, total energy and breastmilk and diffusion tensor imaging metrics of white matter microstructure for the whole cohort.

Predictor: Protein intake (g.kg^{-1}.day^{-1})		FA						MD						AD						RD					
		Days 1-7			Days 1-14			Days 1-7			Days 1-14			Days 1-7			Days 1-14			Days 1-7			Days 1-14		
Predicted variable		Beta	p	Corrected p (FDR)	Beta	p	Corrected p (FDR)	Beta	p	Corrected p (FDR)	Beta	p	Corrected p (FDR)	Beta	p	Corrected p (FDR)	Beta	p	Corrected p (FDR)	Beta	p	Corrected p (FDR)	Beta	p	Corrected p (FDR)
Forceps major		0.003	0.23	0.82	0.002	0.22	0.61	-0.054	0.01	0.05	-0.030	0.01	0.05	-0.040	0.30	0.64	-0.020	0.35	0.95	-0.062	0.03	0.17	-0.036	0.03	0.12
Forceps minor		-0.001	0.62	0.83	0.001	0.24	0.61	-0.016	0.31	0.44	-0.008	0.32	0.62	-0.042	0.14	0.64	0.003	0.85	0.95	-0.003	0.89	0.89	-0.014	0.22	0.43
Anterior thalamic radiation	left	0.001	0.60	0.83	9.4E-5	0.87	0.95	-0.013	0.32	0.44	-0.003	0.71	0.75	-0.014	0.49	0.68	-0.004	0.72	0.95	-0.013	0.33	0.60	-0.002	0.77	0.77
	right	0.001	0.63	0.83	4.1E-5	0.95	0.95	-0.019	0.10	0.27	-0.004	0.51	0.66	-0.023	0.23	0.64	-0.007	0.50	0.95	-0.017	0.17	0.44	-0.003	0.68	0.76
Cingulum angular bundle	left	0.004	0.02	0.20	0.002	0.05	0.23	-0.063	4E-3	0.05	-0.025	0.05	0.27	-0.034	0.32	0.64	-0.009	0.64	0.95	-0.078	3E-4	0.01	-0.033	0.01	0.07
	right	0.004	0.02	0.20	0.002	0.03	0.23	-0.035	0.11	0.27	-0.017	0.15	0.56	-0.001	0.97	0.97	0.003	0.85	0.95	-0.052	0.02	0.17	-0.028	0.03	0.12
Cingulate gyrus	left	-0.001	0.77	0.83	0.001	0.26	0.61	-0.021	0.24	0.44	-0.008	0.40	0.62	-0.043	0.16	0.64	0.006	0.72	0.95	-0.010	0.66	0.80	-0.015	0.20	0.43
	right	-0.001	0.59	0.83	0.001	0.30	0.61	-0.028	0.10	0.27	-0.013	0.18	0.56	-0.062	0.04	0.52	-0.008	0.63	0.95	-0.012	0.54	0.80	-0.015	0.14	0.43
Corticospinal tract	left	0.002	0.19	0.82	3.8E-4	0.56	0.77	-0.016	0.34	0.44	-0.005	0.57	0.66	-0.007	0.77	0.92	-0.004	0.78	0.95	-0.020	0.22	0.50	-0.006	0.51	0.67
	right	-1.0E-4	0.96	0.96	3.4E-4	0.73	0.94	-0.011	0.56	0.63	-0.009	0.37	0.62	-0.022	0.40	0.67	-0.012	0.40	0.95	-0.005	0.82	0.87	-0.008	0.53	0.67
Inferior longitudinal fasciculus	left	-0.001	0.61	0.83	-2.0E-4	0.84	0.95	0.006	0.71	0.71	0.003	0.78	0.78	-0.002	0.94	0.97	0.001	0.96	0.96	0.011	0.61	0.80	0.004	0.75	0.77
	right	0.001	0.56	0.83	0.002	0.05	0.23	-0.040	0.01	0.05	-0.024	4E-3	0.05	-0.045	0.06	0.52	-0.008	0.56	0.95	-0.037	0.05	0.19	-0.032	2E-3	0.04
Superior longitudinal fasciculus parietal	left	3.9E-4	0.79	0.83	0.001	0.34	0.61	-0.008	0.68	0.71	-0.007	0.55	0.66	-0.009	0.71	0.91	-0.003	0.83	0.95	-0.008	0.72	0.80	-0.009	0.46	0.67
	right	-4.0E-4	0.74	0.83	9.1E-5	0.89	0.95	-0.011	0.46	0.55	-0.007	0.42	0.62	-0.019	0.30	0.64	-0.007	0.50	0.95	-0.007	0.67	0.80	-0.007	0.47	0.67
Superior longitudinal fasciculus temporal	left	0.001	0.40	0.83	0.001	0.42	0.63	-0.014	0.32	0.44	-0.004	0.59	0.66	-0.015	0.41	0.67	-0.003	0.78	0.95	-0.014	0.38	0.62	-0.005	0.56	0.67
	right	4.6E-4	0.69	0.83	0.001	0.38	0.62	-0.019	0.20	0.40	-0.011	0.19	0.56	-0.023	0.21	0.64	-0.009	0.39	0.95	-0.017	0.29	0.58	-0.012	0.18	0.43
Uncinate fasciculus	left	0.002	0.05	0.28	0.002	0.01	0.14	-0.018	0.13	0.28	-0.006	0.34	0.62	0.003	0.84	0.95	0.011	0.24	0.95	-0.029	0.04	0.17	-0.015	0.05	0.20
	right	0.001	0.31	0.83	0.001	0.28	0.61	-0.021	0.07	0.27	-0.006	0.37	0.62	-0.013	0.44	0.67	0.001	0.90	0.95	-0.026	0.08	0.25	-0.009	0.25	0.46

Predictor: Fat intake (g.kg^{-1}.day^{-1})		FA						MD						AD						RD					
		Days 1-7			Days 1-14			Days 1-7			Days 1-14			Days 1-7			Days 1-14			Days 1-7			Days 1-14		
Predicted variable		Beta	p	Corrected p (FDR)	Beta	p	Corrected p (FDR)	Beta	p	Corrected p (FDR)	Beta	p	Corrected p (FDR)	Beta	p	Corrected p (FDR)	Beta	p	Corrected p (FDR)	Beta	p	Corrected p (FDR)	Beta	p	Corrected p (FDR)
Forceps major		0.002	0.17	0.32	0.001	0.06	0.24	-0.031	2E-3	0.01	-0.012	0.01	0.08	-0.020	0.32	0.47	-0.001	0.89	0.97	-0.037	0.01	0.04	-0.018	0.01	0.06
Forceps minor		0.002	0.12	0.27	0.001	0.04	0.21	-0.025	2E-3	0.01	-0.007	0.05	0.31	-0.016	0.31	0.47	0.003	0.71	0.93	-0.029	0.01	0.03	-0.012	0.01	0.06
Anterior thalamic radiation	left	2.9E-4	0.63	0.72	-1.8E-4	0.52	0.66	-0.013	0.09	0.12	-0.001	0.83	0.83	-0.017	0.12	0.30	-0.004	0.47	0.93	-0.011	0.15	0.21	0.001	0.85	0.89
	right	-3.0E-4	0.64	0.72	-2.7E-4	0.35	0.49	-0.011	0.09	0.12	-0.001	0.73	0.77	-0.022	0.04	0.23	-0.006	0.23	0.93	-0.006	0.41	0.47	0.001	0.68	0.76
Cingulum angular bundle	left	0.001	0.28	0.46	0.001	0.25	0.45	-0.023	0.07	0.12	-0.008	0.17	0.45	-0.018	0.34	0.47	-0.004	0.67	0.93	-0.025	0.05	0.08	-0.010	0.09	0.19
	right	0.002	0.09	0.25	0.001	0.03	0.21	-0.021	0.08	0.12	-0.006	0.32	0.52	-0.007	0.69	0.78	0.005	0.55	0.93	-0.028	0.02	0.05	-0.011	0.06	0.17
Cingulate gyrus	left	0.003	0.02	0.22	0.002	2E-3	0.03	-0.020	0.04	0.09	-0.006	0.22	0.45	0.004	0.82	0.85	0.013	0.09	0.93	-0.033	0.01	0.03	-0.015	0.01	0.05
	right	0.002	0.10	0.25	0.001	0.24	0.45	-0.022	0.02	0.06	-0.007	0.13	0.45	-0.013	0.44	0.57	-0.004	0.64	0.93	-0.026	0.01	0.04	-0.008	0.08	0.19
Corticospinal tract	left	-6.3E-5	0.92	0.92	1.1E-4	0.72	0.81	-0.011	0.22	0.25	-0.002	0.71	0.77	-0.020	0.15	0.30	-0.001	0.92	0.97	-0.007	0.45	0.47	-0.002	0.62	0.75
	right	-4.7E-4	0.64	0.72	1.1E-4	0.82	0.87	-0.012	0.23	0.25	-0.004	0.44	0.65	-0.028	0.05	0.23	-0.004	0.49	0.93	-0.004	0.72	0.72	-0.003	0.58	0.74
Inferior longitudinal fasciculus	left	1.1E-4	0.91	0.92	-2.6E-5	0.95	0.95	-0.014	0.17	0.20	-0.002	0.66	0.77	-0.021	0.14	0.30	-0.004	0.51	0.93	-0.010	0.41	0.47	-0.001	0.89	0.89
	right	0.001	0.18	0.32	0.001	0.07	0.24	-0.027	1E-3	0.01	-0.012	2E-3	0.04	-0.020	0.13	0.30	-0.005	0.41	0.93	-0.031	4E-3	0.03	-0.015	2E-3	0.04
Superior longitudinal fasciculus parietal	left	0.001	0.08	0.25	5.0E-4	0.18	0.41	-0.022	0.05	0.10	-0.006	0.25	0.45	-0.017	0.22	0.39	-0.003	0.60	0.93	-0.024	0.04	0.07	-0.007	0.17	0.31
	right	4.1E-4	0.56	0.72	1.3E-4	0.67	0.81	-0.020	0.02	0.05	-0.004	0.24	0.45	-0.024	0.02	0.23	-0.004	0.43	0.93	-0.018	0.05	0.08	-0.005	0.25	0.41
Superior longitudinal fasciculus temporal	left	0.001	0.09	0.25	0.001	0.09	0.24	-0.020	0.01	0.04	-0.005	0.21	0.45	-0.017	0.10	0.30	-0.001	0.81	0.97	-0.021	0.01	0.04	-0.006	0.11	0.23
	right	0.001	0.02	0.22	0.001	0.08	0.24	-0.026	1E-3	0.01	-0.006	0.13	0.45	-0.022	0.03	0.23	-0.002	0.73	0.93	-0.028	1E-3	0.02	-0.008	0.05	0.17
Uncinate fasciculus	left	3.8E-4	0.58	0.72	3.0E-4	0.34	0.49	-0.006	0.37	0.37	-0.002	0.54	0.69	-0.006	0.53	0.63	-1.2E-5	1.00	1.00	-0.006	0.44	0.47	-0.003	0.43	0.59
	right	0.002	0.05	0.25	3.9E-4	0.27	0.45	-0.011	0.08	0.12	-0.002	0.47	0.65	0.002	0.85	0.85	0.002	0.71	0.93	-0.018	0.03	0.06	-0.004	0.28	0.42

Predictor: Energy intake (g.kg^{-1}.day^{-1})	FA						MD						AD						RD					
	Days 1-7			Days 1-14			Days 1-7			Days 1-14			Days 1-7			Days 1-14			Days 1-7			Days 1-14		
Predicted variable	Beta	p	Corrected p (FDR)	Beta	p	Corrected p (FDR)	Beta	p	Corrected p (FDR)	Beta	p	Corrected p (FDR)	Beta	p	Corrected p (FDR)	Beta	p	Corrected p (FDR)	Beta	p	Corrected p (FDR)	Beta	p	Corrected p (FDR)

© 2025 Poppe T et al. *JAMA Network Open*.

Forceps major		1.2E-4	0.29	0.52	6.5E-5	0.13	0.23	-0.003	1E-4	2.5E-3	-0.001	2E-3	0.03	-0.003	0.07	0.89	-4.8E-4	0.41	0.98	-0.003	0.01	0.05	-0.001	0.01	0.07
Forceps minor		1.5E-4	0.08	0.19	7.2E-5	0.02	0.13	-0.002	0.01	0.09	-3.6E-4	0.13	0.62	-0.001	0.68	0.89	3.7E-4	0.40	0.98	-0.002	0.01	0.05	-0.001	0.02	0.09
Anterior thalamic radiation	left	3.8E-5	0.39	0.58	-1.9E-6	0.91	0.94	-3.9E-4	0.49	0.72	2.0E-5	0.93	0.98	-1.9E-4	0.81	0.89	-2.4E-5	0.94	0.98	-4.8E-4	0.40	0.55	4.1E-5	0.85	0.90
	right	-4.6E-6	0.92	0.92	-9.9E-6	0.60	0.77	-3.1E-4	0.52	0.72	2.8E-5	0.88	0.98	-0.001	0.43	0.89	-1.3E-4	0.66	0.98	-1.6E-4	0.77	0.81	1.1E-4	0.59	0.71
Cingulum angular bundle	left	1.4E-4	0.05	0.15	4.3E-5	0.13	0.23	-0.001	0.14	0.31	-3.6E-4	0.32	0.65	-1.9E-4	0.89	0.89	5.7E-5	0.92	0.98	-0.002	0.03	0.09	-0.001	0.12	0.27
	right	1.7E-4	0.02	0.10	6.3E-5	0.02	0.13	-4.2E-4	0.64	0.79	-1.6E-4	0.65	0.84	0.002	0.26	0.89	0.001	0.26	0.98	-0.001	0.14	0.22	-0.001	0.14	0.28
Cingulate gyrus	left	1.8E-4	0.04	0.15	9.2E-5	0.01	0.11	-0.001	0.11	0.28	-2.9E-4	0.32	0.65	4.2E-4	0.74	0.89	0.001	0.11	0.98	-0.002	0.03	0.08	-0.001	0.02	0.09
	right	7.0E-5	0.37	0.58	4.1E-5	0.18	0.27	-0.001	0.04	0.14	-3.8E-4	0.17	0.62	-0.002	0.20	0.89	-1.2E-4	0.81	0.98	-0.001	0.09	0.16	-0.001	0.09	0.27
Corticospinal tract	left	2.0E-5	0.69	0.88	6.9E-6	0.72	0.81	-2.6E-4	0.70	0.79	-2.9E-5	0.91	0.98	-1.9E-4	0.85	0.89	7.4E-5	0.85	0.98	-3.0E-4	0.66	0.79	-8.0E-5	0.76	0.86
	right	1.9E-5	0.81	0.91	1.6E-5	0.59	0.77	-3.1E-4	0.69	0.79	-1.6E-4	0.58	0.84	-3.4E-4	0.75	0.89	-5.8E-5	0.89	0.98	-2.9E-4	0.75	0.81	-2.1E-4	0.55	0.70
Inferior longitudinal fasciculus	left	1.6E-5	0.83	0.91	2.3E-6	0.94	0.94	-1.7E-4	0.82	0.82	-4.4E-6	0.99	0.99	-2.4E-4	0.82	0.89	-5.4E-5	0.90	0.98	-1.3E-4	0.88	0.88	2.0E-5	0.95	0.95
	right	1.1E-4	0.11	0.22	5.8E-5	0.04	0.13	-0.002	0.01	0.07	-0.001	0.01	0.06	-0.001	0.41	0.89	-1.2E-4	0.77	0.98	-0.002	0.01	0.05	-0.001	2E-3	0.04
Superior longitudinal fasciculus parietal	left	1.3E-4	0.03	0.15	4.1E-5	0.08	0.21	-0.001	0.20	0.33	-3.2E-4	0.33	0.65	-2.0E-4	0.85	0.89	-2.4E-5	0.95	0.98	-0.002	0.08	0.16	-4.6E-4	0.17	0.31
	right	9.4E-6	0.86	0.91	8.6E-6	0.67	0.80	-0.001	0.19	0.33	-2.2E-4	0.36	0.65	-0.001	0.18	0.89	-1.6E-4	0.60	0.98	-0.001	0.30	0.45	-2.5E-4	0.35	0.48
Superior longitudinal fasciculus temporal	left	1.2E-4	0.01	0.10	3.5E-5	0.07	0.21	-0.001	0.05	0.14	-2.6E-4	0.27	0.65	-3.8E-4	0.63	0.89	7.9E-6	0.98	0.98	-0.002	0.01	0.05	-3.9E-4	0.12	0.27
	right	1.3E-4	0.01	0.10	3.9E-5	0.04	0.13	-0.001	0.03	0.14	-3.4E-4	0.17	0.62	-4.1E-4	0.59	0.89	1.8E-5	0.95	0.98	-0.002	0.01	0.05	-0.001	0.05	0.17
Uncinate fasciculus	left	4.1E-5	0.42	0.58	3.0E-5	0.12	0.23	-1.5E-4	0.76	0.81	-9.4E-5	0.64	0.84	1.9E-4	0.78	0.89	1.8E-4	0.52	0.98	-3.2E-4	0.59	0.75	-2.3E-4	0.32	0.48
	right	1.1E-4	0.06	0.16	3.0E-5	0.18	0.27	-0.001	0.19	0.33	-1.2E-4	0.52	0.84	4.1E-4	0.57	0.89	1.9E-4	0.49	0.98	-0.001	0.06	0.13	-2.8E-4	0.24	0.40

Predictor: Breastmilk intake (ml.kg^{-1}day^{-1})		FA						MD						AD						RD					
		Days 1-7			Days 1-14			Days 1-7			Days 1-14			Days 1-7			Days 1-14			Days 1-7			Days 1-14		
Predicted variable		Beta	p	Corrected p (FDR)	Beta	p	Corrected p (FDR)	Beta	p	Corrected p (FDR)	Beta	p	Corrected p (FDR)	Beta	p	Corrected p (FDR)	Beta	p	Corrected p (FDR)	Beta	p	Corrected p (FDR)	Beta	p	Corrected p (FDR)
Forceps major		2.4E-5	0.64	0.82	1.7E-5	0.29	0.76	-0.001	0.04	0.11	-2.9E-4	0.01	0.12	-0.001	0.25	0.45	-2.0E-4	0.38	0.74	-0.001	0.17	0.24	-3.3E-4	0.05	0.22
Forceps minor		3.3E-5	0.37	0.62	8.3E-6	0.49	0.76	-0.001	0.02	0.11	-1.2E-4	0.17	0.34	-4.9E-4	0.33	0.45	-6.5E-5	0.70	0.74	-0.001	0.05	0.13	-1.5E-4	0.21	0.37
Anterior thalamic radiation	left	1.6E-5	0.41	0.62	-1.5E-6	0.82	0.93	-3.7E-4	0.14	0.19	-3.8E-5	0.66	0.72	-3.9E-4	0.29	0.45	-7.8E-5	0.53	0.74	-3.7E-4	0.15	0.23	-1.8E-5	0.83	0.89
	right	-7.9E-6	0.71	0.86	-5.0E-6	0.49	0.76	-2.9E-4	0.18	0.23	-2.5E-5	0.74	0.74	-0.001	0.10	0.33	-1.1E-4	0.36	0.74	-1.5E-4	0.52	0.60	1.8E-5	0.82	0.89
Cingulum angular bundle	left	2.9E-5	0.37	0.62	6.1E-6	0.59	0.76	-0.001	0.22	0.26	-1.4E-4	0.33	0.49	-3.5E-4	0.58	0.70	-1.0E-4	0.64	0.74	-0.001	0.15	0.23	-1.6E-4	0.27	0.44
	right	4.6E-5	0.15	0.48	1.2E-5	0.28	0.76	-0.001	0.08	0.15	-1.9E-4	0.16	0.34	-3.4E-4	0.57	0.70	-8.9E-5	0.67	0.74	-0.001	0.03	0.13	-2.5E-4	0.08	0.22
Cingulate gyrus	left	6.7E-5	0.08	0.48	2.4E-5	0.07	0.76	-0.001	0.10	0.16	-1.2E-4	0.30	0.49	8.1E-5	0.89	0.89	1.4E-4	0.46	0.74	-0.001	0.03	0.13	-2.5E-4	0.07	0.22
	right	4.9E-5	0.16	0.48	8.4E-6	0.48	0.76	-0.001	0.08	0.15	-1.6E-4	0.13	0.34	-2.4E-4	0.67	0.76	-1.4E-4	0.49	0.74	-0.001	0.04	0.13	-1.8E-4	0.13	0.27
Corticospinal tract	left	-1.1E-5	0.62	0.82	9.1E-7	0.90	0.96	-3.4E-4	0.26	0.29	-7.1E-5	0.50	0.64	-0.001	0.10	0.33	-9.7E-5	0.53	0.74	-1.5E-4	0.63	0.66	-5.8E-5	0.58	0.74
	right	-4.2E-5	0.21	0.48	-8.1E-6	0.48	0.76	-2.3E-4	0.50	0.50	-5.6E-5	0.63	0.72	-0.001	0.05	0.33	-1.9E-4	0.24	0.74	1.2E-4	0.77	0.77	1.2E-5	0.93	0.93
Inferior longitudinal fasciculus	left	1.1E-6	0.97	0.97	-8.0E-8	0.99	0.99	-4.8E-4	0.14	0.19	-4.6E-5	0.68	0.72	-0.001	0.11	0.33	-8.6E-5	0.59	0.74	-3.4E-4	0.39	0.50	-2.6E-5	0.84	0.89
	right	2.6E-5	0.41	0.62	6.8E-6	0.53	0.76	-0.001	0.03	0.11	-2.4E-4	0.01	0.12	-4.7E-4	0.29	0.45	-2.3E-4	0.12	0.74	-0.001	0.06	0.13	-2.5E-4	0.04	0.22
Superior longitudinal fasciculus parietal	left	3.4E-5	0.21	0.48	2.8E-6	0.76	0.91	-0.001	0.08	0.15	-1.3E-4	0.29	0.49	-0.001	0.23	0.45	-1.4E-4	0.37	0.74	-0.001	0.06	0.13	-1.3E-4	0.32	0.48
	right	5.9E-6	0.80	0.90	4.2E-6	0.59	0.76	-0.001	0.03	0.11	-1.7E-4	0.06	0.28	-0.001	0.03	0.33	-1.8E-4	0.12	0.74	-4.8E-4	0.11	0.20	-1.7E-4	0.10	0.22
Superior longitudinal fasciculus temporal	left	2.8E-5	0.20	0.48	1.0E-5	0.18	0.76	-0.001	0.03	0.11	-1.3E-4	0.15	0.34	-4.9E-4	0.16	0.42	-6.7E-5	0.57	0.74	-0.001	0.03	0.13	-1.6E-4	0.09	0.22
	right	3.9E-5	0.07	0.48	1.2E-5	0.09	0.76	-0.001	0.01	0.11	-1.9E-4	0.05	0.27	-0.001	0.08	0.33	-1.1E-4	0.35	0.74	-0.001	0.01	0.13	-2.3E-4	0.02	0.22
Uncinate fasciculus	left	2.2E-6	0.92	0.97	4.8E-6	0.54	0.76	-2.2E-4	0.34	0.36	-6.8E-5	0.38	0.53	-3.2E-4	0.31	0.45	-4.8E-5	0.66	0.74	-1.7E-4	0.54	0.60	-7.8E-5	0.39	0.55
	right	3.9E-5	0.13	0.48	1.1E-5	0.20	0.76	-3.9E-4	0.08	0.15	-1.1E-4	0.14	0.34	-5.1E-5	0.88	0.89	-3.8E-6	0.97	0.97	-0.001	0.04	0.13	-1.6E-4	0.08	0.22

FA = fractional anisotropy, MD = mean diffusivity, AD = axial diffusivity, RD = radial diffusivity. Linear regression models were adjusted for sex, birthweight z-score, New Zealand socioeconomic deprivation index. All analyses except for those involving metrics of Total Intracranial Volume, Relative Brain Volume to Intracranial Volume and Brain Volume were corrected for brain volume.

© 2025 Poppe T et al. *JAMA Network Open*.
