# Supplementary material for: Neonatal Nutrition and Brain Structure at 7 Years in Children Born Very Preterm
Source: JAMA Netw Open. 2025 Jan 24;8(1):e2456080. doi: 10.1001/jamanetworkopen.2024.56080 (PMC11762234; doi:10.1001/jamanetworkopen.2024.56080)
Supplement: Supplement 2. — Nonauthor Collaborators [file jamanetwopen-e2456080-s002.pdf]

Supplemental Online Content: Nonauthor Collaborators
*First name, last name, and suffix (if applicable) are required and will appear in PubMed.

***Group Name(s): PIANO Study Group**

***First Name and Middle Initial(s)**	***Last Name**	***Suffix (eg, Jr, III)**	Academic Degrees	Institution	Location (city, state/province, country)	Role or Contribution, eg, chair, principal investigator	Group (if more than 1 Group listed in the byline) and/or Subgroup (eg, Steering Committee)
Janene B	Biggs		BNurs	University of Auckland	Auckland, New Zealand	Investigator	
Coila	Bevan		MSc	University of Auckland	Auckland, New Zealand	Investigator	
Joanna M	Black		PhD	University of Auckland	Auckland, New Zealand	Investigator	
Frank H	Bloomfield		PhD	University of Auckland	Auckland, New Zealand	Investigator	
Kelly	Fredell		BNurs	University of Auckland	Auckland, New Zealand	Investigator	
Sabine	Huth		BA	University of Auckland	Auckland, New Zealand	Investigator	
Christine	Keven		NZCS	University of Auckland	Auckland, New Zealand	Investigator	
Myra	Leung		PhD	University of Canberra	Canberra, ACT, Austrailia	Investigator	
Geraint	Phillips		OD	University of Auckland	Auckland, New Zealand	Investigator	
Jennifer A	Rogers		MSc	University of Auckland	Auckland, New Zealand	Investigator	
Heather	Stewart		BSc	University of Auckland	Auckland, New Zealand	Investigator	
Kathryn A	Williamson		MSc	University of Auckland	Auckland, New Zealand	Investigator	
Trecia A	Wouldes		PhD	University of Auckland	Auckland, New Zealand	Investigator	
