# Supplementary material for: Neonatal Nutrition and Brain Structure at 7 Years in Children Born Very Preterm
Source: JAMA Netw Open. 2025 Jan 24;8(1):e2456080. doi: 10.1001/jamanetworkopen.2024.56080 (PMC11762234; doi:10.1001/jamanetworkopen.2024.56080)
Supplement: Supplement 3. — Data Sharing Statement [file jamanetwopen-e2456080-s003.pdf]

# Data Sharing Statement

Poppe. Neonatal Nutrition and Brain Structure at 7 Years in Children Born Very Preterm. *JAMA Netw Open*. Published January 24, 2025. doi:10.1001/jamanetworkopen.2024.56080

**Data**
**Data available:** No

**Additional Information**
**Explanation for why data not available:** We do not have ethical approval to share the study data
